# Investigation of the Variants of Independent Elastic Constants of Rigid Polyurethane Foams with Symmetry Elements

**DOI:** 10.3390/polym17172431

**Published:** 2025-09-08

**Authors:** Aivars Lagzdiņš, Ilze Beverte, Vilis Skruls, Jānis Andersons

**Affiliations:** Institute for Mechanics of Materials, University of Latvia, 3 Jelgavas St., LV-1004 Riga, Latvia; aivars.lagzdins@gmail.com (A.L.); skruls@pmi.lv (V.S.)

**Keywords:** polyurethane foams, linear elasticity, symmetry, transtropy, independent elastic constants, variants, uncertainties

## Abstract

Rigid PU foams have wide practical applications, and their mathematical modelling would benefit from deeper knowledge about the variants of independent elastic constants of symmetric PU foams. Therefore, in this study, various symmetry elements of rigid PU foams were analysed in relation to the characteristics of production moulds and technologies. The generalised Hooke’s law was considered together with additional relationships valid for certain types of symmetry. Variants of independent elastic constants were determined for orthotropic, orthotropic with a rotational symmetry, and isotropic PU foams. For transtropic PU foams, nine variants of independent elastic constants were identified and corresponding equations for the components of response strain tensor were derived. Then, in order to investigate the results provided by the 9 variants, 12 elastic constants were determined experimentally in compression and shear for free-rise, rigid, and quasi-transtropic PU foams with average densities of 34 kg/m^3^, 55 kg/m^3^, and 75 kg/m^3^. Based on the analysis of (a) measurement uncertainties and (b) satisfying of the transtropy equations, an assessment was made of the correspondence of the experimentally determined elastic constants to the constants of a perfectly transtropic material. This made it possible to identify variants of independent constants that ensure the best correspondence between the calculated strains and the set of average strains.

## 1. Introduction

Rigid polyurethane (PU) foams are polymer–gas composites with outstanding thermal insulation properties and acceptable load bearing capacity, which are applied in various engineering solutions [1,2]. The foams are used in the construction sector in sandwich structures and panels; as thermal insulation of mortars for masonry; in the transport sector in cooling vans and vehicle insulation; for both heat and cold saving insulation in stores, warehouse buildings, and constructions; for the thermal insulation of cryogenic tanks in space vehicles; as emergency shock absorbers; as electric insulation in electronics, equipment housing, and components; in radomes (protective structures for out-door antennas, locators and telescopes); for providing a radio-frequency transparent layer along dimensional stability, etc. [3,4]. Practical applications and mathematical modelling require knowledge on the symmetry of PU foams’ structure and physical/mechanical properties.

Anisotropic materials are usually classified by their symmetry properties with respect to orthogonal transformations of the coordinate system [5,6]. For PU foams, produced by foaming of the liquid chemical formulation in a mould, the symmetry of the moulded blocks is determined mainly by the characteristics of the mould: its absolute and relative dimensions, shape (rectangular, circular cylindrical, conical), open or sealed [3,4]. A study of cellular structure of rigid PU foams in moulded free-rise rectangular blocks (dimensions 200 cm × 100 cm × 30 cm) by cutting cubical samples (size 5 cm × 5 cm × 5 cm) from the block is described in [3]. The foams were found to have nearly spherical cells in the centre of the blocks (quasi-isotropic) and elongated cells, orientated mainly in rise direction, near the walls of the mould.

The effect of processing temperature and mould size (aluminium cylinders with diameters of 29 mm, 41 mm, and 51 mm) on the average density and density gradients (radial and vertical) for free-rise, water blown, and rigid polyurethane foam systems was studied in [7]. Both average density and radial density gradients decreased with increasing processing temperature and larger mould sizes. In the study [8], rigid transtropic PU foams were produced in free-rise process, in relatively high, circular cylindrical aluminium moulds with an inner diameter of ≈29 mm and height (a) ≈140 mm and (b) ≈457 mm, as well as in conical moulds. Microscopy revealed transtropy in the central part of the cylindrical PU foams’ samples, while general anisotropy was identified near the wall of the metallic mould.

Symmetry elements of rigid PU foams blocks, produced in delimited foaming in a rectangular, sealed mould [9], are discussed in [10]. The blocks were shaped as a truncated pyramid, with top dimensions of 15 cm × 15 cm, bottom of 14 cm × 14 cm, and a height of 5 cm. The fourth-order rotational symmetry around the rise direction was identified for blocks produced in the mould with square cross-section, which facilitates forecasting of locations with similar foaming conditions in the block.

Neumann’s principle can be extended to symmetric PU foams; when the structure of a material is invariant with respect to certain symmetry elements, any of its physical properties must be invariant with respect to at least the same symmetry elements [11,12]. The general equations of linear elasticity and independent elastic constants are discussed in [12,13] for the main kinds of symmetry. Since components of stiffness and compliance tensors have to be invariant at symmetry transformations of a material, reduction in the number of independent elastic constants due to symmetry is analysed in [14,15].

Representation theory [16,17] deals with the effect of symmetries on solutions of equations and behaviour of objects. When symmetry elements of a certain medium form a finite group, the number of independent components of compliance tensor s_ijkl_ can be determined by means of linear representation theory of groups [18]. In [18], the independent components of the elasticity tensors of fourth- to twelfth-order are derived for several kinds of anisotropic media. One of the variants of five independent elastic constants is considered for rigid transtropic (montropic) PU foams in [19]. In [20], calculation of the dependent constants of transtropic plastics foams with pronounced strut-like structure is considered for the same variant of independent constants. In [21], equations are derived expressing a variant of five independent moduli of transtropic light-weight foams, but the relationships remain in too general a form, which complicates practical application. In [22], an advanced mechanical characterisation of one of the variants of elastic constants of orthotropic PU foams is carried out. In [23], the numerical values of the same variant of stiffness tensor are calculated numerically for orthotropic foams using finite element simulations and ANN, but other variants are not considered.

An analysis of scientific information sources revealed a limited number of theoretical studies relevant to determining variants of independent elastic constants of PU foams with symmetry elements. The methodology for deriving the variants is not clearly outlined. A shortage of experimental data for various variants of constants was identified as well.

This study aimed to determine variants of independent elastic constants for rigid-moulded PU foams with symmetry elements. The generalised Hooke’s law is considered in a Cartesian coordinate system, together with additional relationships valid for different types of symmetry. Nine variants of independent elastic constants are identified for the wide-spread transtropic PU foams and corresponding equations are derived for the response strain components. The elastic constants are determined experimentally for quasi-transtropic PU foams with average densities of 34 kg/m^3^, 55 kg/m^3^, and 75 kg/m^3^ and the variants of independent constants, which provide the best correspondence with the constants of perfectly transtropic PU foams, are outlined.

## 2. Theoretical Part

### 2.1. Orthotropic PU Foams

Anisotropic rigid PU foams can be obtained by spray-on technology [3,4,24,25]. For anisotropic foams, the generalised Hooke’s law in an ortho-normal coordinate system X_i_, i = 1, 2, 3 is written as ε_ij_ = s_ijkl_σ_kl_, where ε_ij_ is the strain tensor, σ_ij_ is the stress tensor, and s_ijkl_ is the fourth-rank tensor of elastic compliance; i, j, k, l = 1, 2, 3 [13,14]. An elastic material with stress and strain symmetry has 21 independent elastic constants, since s_ijkl_ = s_jikl_ = s_ijlk_ = s_jilk_.

Homogeneous blocks of rigid PU foams with certain symmetry elements are produced in rectangular or circular, cylindrical moulds, in free or delimited foaming [3,8,9]. Furthermore, it is assumed that the transversal dimensions of a mould are large compared to the dimensions of the foams’ structural elements. Only that part of the moulded blocks is considered which is sufficiently far from the walls of a block, where contact with the mould leads to un-adiabatic processes and inhomogeneous structure.

**Figure 1:**
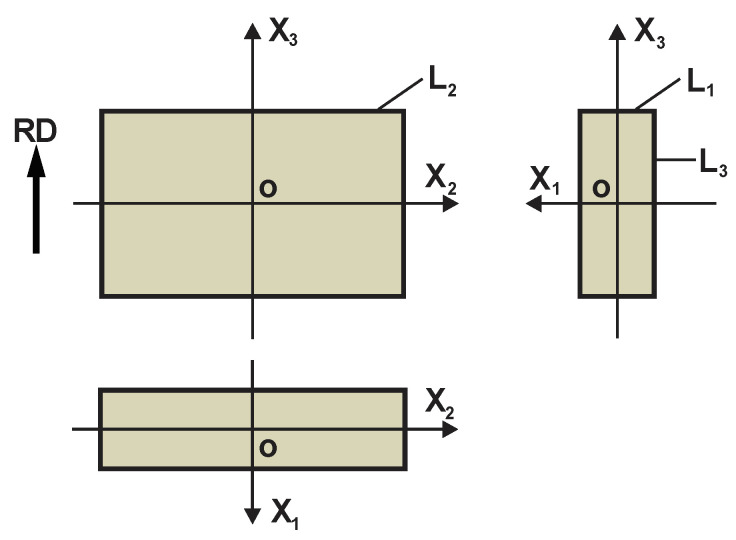
Dimensions of a mould for production of rigid, free-rise, orthotropic PU foams; RD—rise direction (a general scheme; front, side, and top views).

Orthotropic PU foams are produced in free-rise, in a high, long, and narrow rectangular mould, whose height and one transversal dimension significantly exceed the other transversal one: L_2_, L_3_ >> L_1_ and L_3_ > L_2_ (Figure 1).

Orthotropic PU foams have three mutually orthogonal symmetry planes: X_1_OX_2_, X_2_OX_3_, and X_1_OX_3_. Directing the coordinate axis OX_1_, OX_2_, and OX_3_ perpendicular to each plane, the generalised Hooke’s law in tensor notations is written as [12,13,14]

ε_11_ = s_1111_σ_11_ + s_1122_σ_22_ + s_1133_σ_33_,  ε_22_ = s_2211_σ_11_ + s_2222_σ_22_ + s_2233_σ_33_, ε_33_ = s_3311_σ_11_ + s_3322_σ_22_ + s_3333_σ_33_, ε_23_ = 2s_2323_σ_23_,         ε_13_ = 2s_1313_σ_13,_ and       ε_12_ = 2s_1212_σ_12_.          (1)

Using the engineering constants—the elastic moduli E_i_, shear moduli G_ij_, and Poisson’s ratios ν_ij_, i and j = 1, 2, and 3—Equation (1) becomes

(2) $$\begin{array}{l} \varepsilon_{11}=\frac{1}{E_{1}}\sigma_{11}-\frac{\nu_{21}}{E_{2}}\sigma_{22}-\frac{\nu_{31}}{E_{3}}\sigma_{33},\\ \varepsilon_{22}=-\frac{\nu_{12}}{E_{1}}\sigma_{11}+\frac{1}{E_{2}}\sigma_{22}-\frac{\nu_{32}}{E_{3}}\sigma_{33},\\ \varepsilon_{33}=-\frac{\nu_{13}}{E_{1}}\sigma_{11}-\frac{\nu_{23}}{E_{2}}\sigma_{22}+\frac{1}{E_{3}}\sigma_{33},\\ \varepsilon_{23}=\frac{1}{{2G}_{23}}\tau_{23},\\ \varepsilon_{13}=\frac{1}{{2G}_{13}}\tau_{13},\;\mathrm{and}\\ \varepsilon_{12}=\frac{1}{{2G}_{12}}\tau_{12}. \end{array}$$

In Poisson’s ratios, $\nu_{ij}=-\frac{\varepsilon_{jj}}{\varepsilon_{ii}}$, the first index corresponds to the direction of applied stress and the second to that of response strain; ν_ij_ ≠ ν_ji_. In denotations of tangential stresses τ_ij_ and shear moduli G_ij_, the first index corresponds to the plane of action of the applied stress and the second to its directions; G_ji_ = G_ij_. Equations (2) comprise 12 constants: 3 moduli, E_1_, E_2_, E_3_; 6 Poisson’s ratios, ν_21_, ν_12_, ν_31_, ν_13_, ν_32_, ν_23_; and 3 shear moduli, G_23_, G_13_, G_12_. Owing the symmetry of compliance coefficients s_iijj_ = s_jjii_ in Equation (1), we obtain s_1122_ = s_2211_, s_1133_ = s_3311_, and s_2233_ = s_3322_. Then, it follows from (2)

(3) $$\frac{\nu_{ij}}{E_{i}}=\frac{\nu_{ji}}{E_{j}};\;i,\;j=1,\;2\;\mathrm{and}\;3;\;i\neq j;\;\frac{\nu_{21}}{E_{2}}=\frac{\nu_{12}}{E_{1}},\frac{\nu_{31}}{E_{3}}=\frac{\nu_{13}}{E_{1}}\;\mathrm{and}\;\frac{\nu_{32}}{E_{3}}=\frac{\nu_{23}}{E_{2}}.$$

and there remain 12 − 3 = 9 independent constants. The moduli G_23_, G_13_, G_12_, E_1_, E_2_, and E_3_ cannot be expressed by the other constants in any way (each modulus E_1_, E_2_, and E_3_ is comprised in two equations of Equations (3)). Considering all possible combinations of constants and taking into account Equations (3), eight variants of nine independent constants were identified (Table 1).

### 2.2. Symmetry to the Rotation Angle

**Figure 2:**
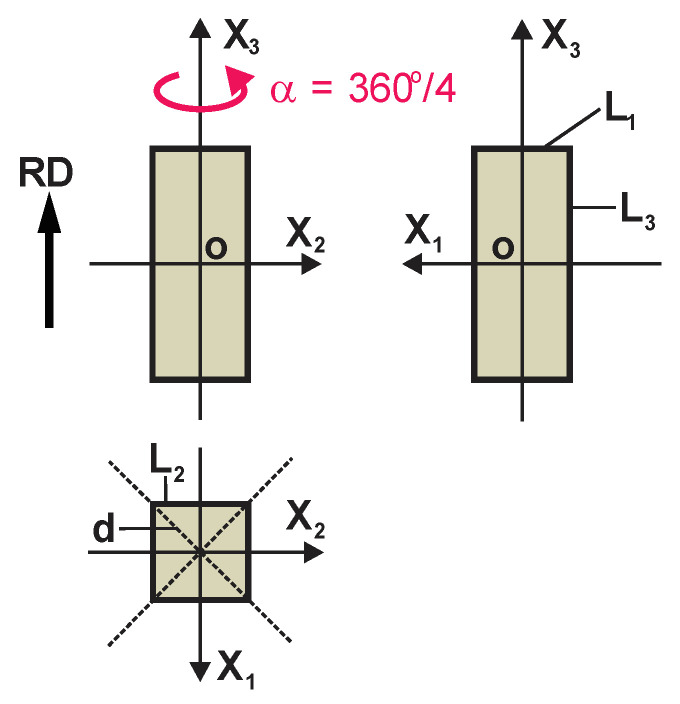
Dimensions of a mould for production of rigid, free-rise, orthotropic PU foams, symmetrical to the rotation angle α = 360°/4 = 90° about the OX_3_ axis.

When PU foams are produced in the free-rise, in a high mould of equal transversal dimensions of the rectangular cross-section L_3_ >> L_1_, L_2_ and L_1_ = L_2_, then, strictly speaking, the foams in the moulded block cannot be considered as perfectly transtropic, since the diagonal of the cross-section of mould is longer than the side: d = $\sqrt{2}$L_2_ (Figure 2).

Only in the central part of the mould can a transtropic structure of foam be expected. The size and shape of the transtropic part has to be estimated individually for each mould and PU foam’s formulation. At the same time, an additional element of structural and elastic symmetry appears; namely, fourth-order rotational symmetry with respect to the rotation angle α = 360°/4 about the OX_3_ axis (Figure 2). Then, the additional relationships E_1_ = E_2_ and G_23_ = G_13_ are valid. From Equations (3), we obtain that

(4) $$\frac{\nu_{21}}{E}=\frac{\nu_{12}}{E},i.e.,\nu_{21}=\nu_{12};\;\nu_{31}=\nu_{32},\;\mathrm{and}\;\nu_{13}=\nu_{23}.$$

Taking into account Equations (4), the following notations are further adopted for simplicity: E_1_ = E_2_ = E, E_3_ = E′; ν_21_ = ν_12_ = ν, ν_31_ = ν_32_ = ν′, ν_13_ = ν_23_ = ν″; G_23_ = G_32_ = G_31_ = G_13_ = G′; and G_12_ = G_21_ = G. Here, E and E′ are Young’s moduli under uniaxial compression/tension in the direction of the plane of isotropy and perpendicular to it, ν is Poisson’s ratio characterising transverse compression/tension in the plane of isotropy under tension/compression in the same plane, ν′ is Poisson’s ratio characterising transverse compression/tension in the plane of isotropy under tension/compression in direction perpendicular to the plane of isotropy, and ν″ is Poisson’s ratio characterising transverse compression/tension in the plane perpendicular to the plane of isotropy under tension/compression in the plane of isotropy [12,13,14]. Then, implementing the new notations, Equations (2) takes the form

(5) $$\begin{array}{l} \varepsilon_{11}=\frac{1}{E}{(\sigma }_{11}-\nu \sigma_{22})-\frac{\nu^{\prime }}{E^{\prime }}\sigma_{33},\\ \varepsilon_{22}=\frac{1}{E}(-\nu \sigma_{11}+\sigma_{22})-\frac{\nu^{\prime }}{E^{\prime }}\sigma_{33},\\ \varepsilon_{33}=\frac{\nu^{\prime \prime }}{E}\left(-\sigma_{11}-\sigma_{22}\right)+\frac{1}{E^{\prime }}\sigma_{33},\\ \varepsilon_{23}=\frac{1}{2G^{\prime }}\tau_{23},\\ \varepsilon_{13}=\frac{1}{2G^{\prime }}\tau_{13},\mathrm{and}\\ \varepsilon_{12}=\frac{1}{2G}\tau_{12}. \end{array}$$

Due to the symmetry of compliance coefficients s_1133_ = s_3311_, from Equations (5), we obtain

(6) $$\frac{\nu^{\prime \prime }}{E}=\frac{\nu^{\prime }}{E^{\prime }}.$$

Altogether, Equations (5) comprises seven constants: E, E′, ν, ν′, v’’, and G, G′. The constants G′ and ν, G are independent, since they cannot be expressed by the other ones, but E, E; ν′, and v’’ can be expressed from (6) in four variants:

(7) $$E^{\prime }=E\frac{\nu^{\prime }}{\nu^{\prime \prime }},\;E=E^{\prime }\frac{\nu^{\prime \prime }}{\nu^{\prime }},\;\nu^{\prime }=E^{\prime }\frac{\nu^{\prime \prime }}{E},\;\mathrm{and}\;\nu^{\prime \prime }=E\frac{\nu^{\prime }}{E^{\prime }}.$$

Thus, four variants of six independent constants have been determined for PU foams with a fourth-order rotational symmetry axis OX_3_: (1) G′, G, E, ν, ν′, ν″; (2) G′, G, E′, E, ν, ν″; (3) G′, G, E′, ν, ν″, ν′; and (4) G′, G, E, E′, ν, ν′.

### 2.3. Transtropic PU Foams

If PU foams are symmetrical to an arbitrary rotation angle α about the OX_3_ axis, the order of rotational symmetry is infinite and the foams are transtropic with the plane of isotropy X_1_OX_2_ and monotropy axis OX_3_. Then, the following relationship is valid:

(8) $${G=G}_{12}=\frac{E_{1}}{2(1+\nu_{12})}.$$

**Figure 3:**
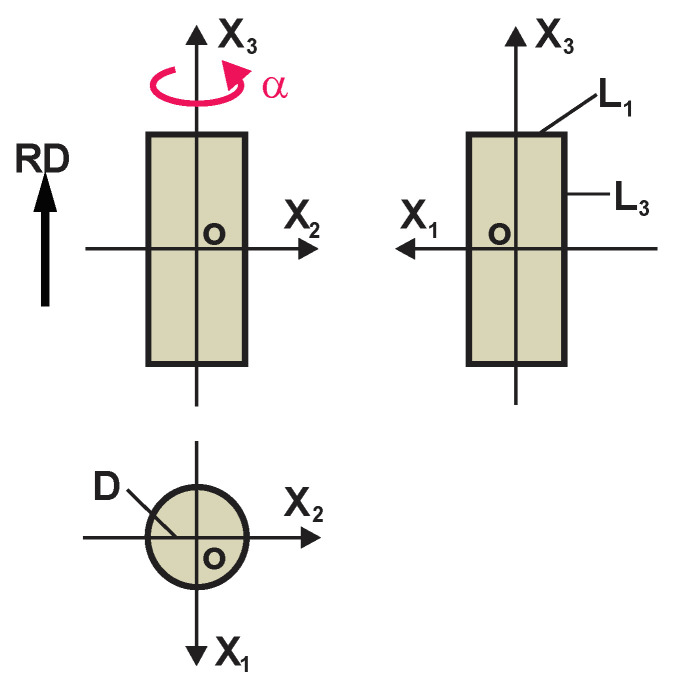
Dimensions of a mould for production of rigid, free-rise, transtropic PU foams.

Equation (8), together with Equations (7), permits the reduction of the number of independent constants to five in Equations (5). Perfectly transtropic PU foams can be produced free-rise, in a comparatively high, cylindrical mould with constant transversal dimensions: L_3_ >> L_1_, L_2_ and L_1_ = L_2_ = D, where D is the diameter of cross-section of the mould [7,8] (Figure 3).

Let us estimate how many variants of the five independent elastic constants can exist for transtropic PU foams. With this aim, we consider the constants E, E′, ν, ν′, ν″ and G, G′ of Equations (5). Obviously, the shear modulus G′ has to be present in all variants, since it cannot be expressed by other constants. E, G, and ν can be expressed by other ones in three variants from Equation (8):

(9) $$G=\frac{E}{2(1+\nu )},\;E\;=\;2G(1\;+\;\nu )\;\mathrm{and}\;\nu =\frac{E}{2G}-1.$$

E′, E″, ν′, and ν″ can be expressed via each other in four variants, given in Equations (7). Variants of constants are analysed in Table S1 of the Supplementary Materials, where the sub-variants from columns 1, 2, and 3 are summarised in column 4. When there were five constants in a variant, it was written in engineering constants with indices in the column 5 and an ordinal number “n” was assigned to it in the column 6. If there were six constants in the variant, constants from column 3 were considered one by one for expression via Equations (7). When the resulting variant comprised five constants, it was compared with the numbered variants, which were identified already previously. When the variant was new, it was written in engineering constants with indices and a number was assigned to it. When the variant coincided with an already identified one, it was omitted. If the resulting variant still had six elements, it was omitted as well. Altogether, nine different variants were identified (Table 2).

Strain components ε_ij_ of the system of Equations (5) were expressed via the independent constants of each variant Equations (S1)–(S9) of the Supplementary Materials). The strain tensors (S1)–(SS9) fully describe the stress–strain state of transtropic PU foams in the principal coordinate system.

### 2.4. Isotropic PU Foams

When all directions in a PU foam’s material are elastically equivalent and principal, the foams are isotropic. Then, E′ = E, ν′ = ν″ = ν; G′ = G = E/[2 (1 + ν)], and E, ν, and G can be expressed via each other in three variants, which provides three different variants of independent constants: (1) E, ν, (2) G, ν, and (3) E, G.

**Figure 4:**
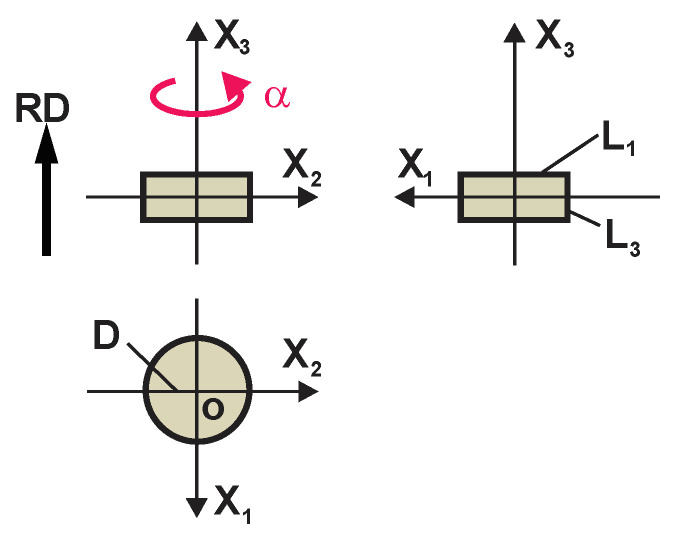
Dimensions of a cylindrical mould for production of rigid, free-rise, isotropic PU foams.

**Figure 5:**
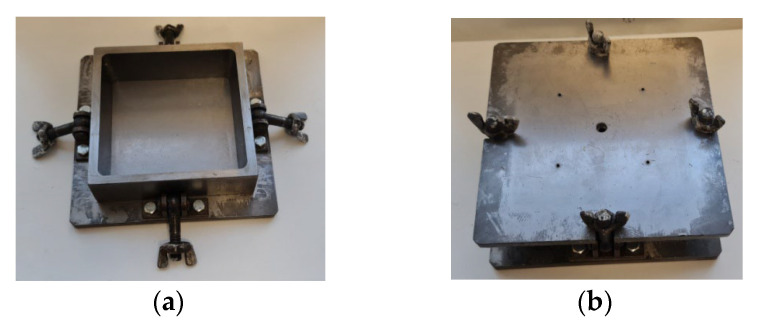
A sealable mould (**a**) open and (**b**) with lid sealed.

The free-rise technology causes some elongation of PU foams’ cells parallel to the rise direction and the foams produced are transtropic to some degree [3,7,8,9]. Quasi-isotropic PU foams can be produced in the free-rise, in a low, cylindrical mould of dimensions L_1_ = L_2_ = R and L_3_ ≤ L_1_, L_2_; Figure 4. Perfectly isotropic PU foams are obtained in foaming under an overpressure, in a sealable mouldmold, Figure 5 [10].

Practically, the central part of any PU foam’s block, produced in the free-rise, in sufficiently low and large mould of equal transversal dimensions can be considered as quasi-isotropic.

## 3. Materials and Experimental Methods

Three rectangular blocks of rigid, free-rise, core PU foams of standard petrochemical formulation, with dimensions of 500 mm × 600 mm × 200 mm and average densities of 34 kg/m^3^, 55 kg/m^3^, and 75 kg/m^3^ were acquired from Bayer AG (Leverkusen, Germany). The geometric centre “O” of each block was determined as the crossing point of the spatial diagonals (Figure S1 of Supplementary Materials). Two parallelepipeds, located on either side of the geometric centre and the plane X_1_OX_3_, were cut from each block: N1 (200 mm × 150 mm × 100 mm) for compression samples and N2 (100 mm × 122 mm × 100 mm) for shear samples (Figures S2 and S3 of Supplementary Materials). Compression and shear samples were made with a band saw, with seam allowances of 1–2 mm; the samples were then sanded to the final dimensions.

To ensure a homogeneous stress field in the measurement zone, the uniaxial compression samples were made as rectangular prisms of dimensions 50 mm × 50 mm × 100 mm [3,26] (Figure S2 of Supplementary Materials). The experimental setup and measurements were carried out according to methodology given in [26]. Deformation parallel to the loading direction was measured with a clip-on extensometer (MTS Model 632.11C-20 type; MTS Systems Corporation, Eden Prairie, MN, USA) attached in the middle of sample’s height, on a measurement base of 10 mm, and with a strain rate 10%/min. The deformations perpendicular to the loading direction were measured with two similar extensometers on the sides of sample’s cross-section [26]. The elastic constants E_1_, ν_21_, ν_31_, E_2_, ν_12_, ν_32_, E_3_, ν_13_, and ν_23_ were determined from the stress–strain curves according to ISO 844:2021.

The shear samples were made as rectangular prisms with dimensions of 14 mm × 22 mm × 100 mm (Figure S3 of Supplementary Materials). Deformation was measured in the centre of a sample’s side, on a 6 mm × 38 mm measurement base, at a strain rate of 3.4 mm/min (9%/min) [3,27]. The experimental setup and measurements were carried out according to methodology given in [27]. Constants G_12_, G_13_, and G_23_ were determined from corresponding stress–strain curves according to the standard ASTM C273/C273M.

The density of all samples was determined according to ISO 845:2006. Both in compression and in shear, four samples were tested under reproducibility conditions and at ambient temperatures of 20 °C ≤ T ≤ 22 °C and relative humidity of 49% ≤ RH ≤ 53%; no conditioning was made for the samples. All samples were tested on the electromechanical testing machine 2166P-5 which used the automated data-gathering system Spider v1.22. Tangents were drawn to the uniaxial compression curves “σ_11_–ε_11_”, “σ_22_–ε_22_”, and “σ_33_–ε_33_”, as well as to the shear curves “τ_12_–ε_12_”, “τ_13_–ε_13_”, and “τ_23_–ε_23_” in the elastic region, in order to determine the elastic constants (Figures S4 and S5 of the Supplementary Materials). The limit stresses σ_11_^lim^, σ_22_^lim^, and σ_33_^lim^, as well as τ_12_^lim^, τ_13_^lim^, and τ_23_^lim^, were calculated as the stresses at which the curves deviated from tangents.

## 4. Numerical Calculations

### 4.1. The Elastic Constants

The strains ε_ij_, corresponding to the nine variants of independent elastic constants of transtropic PU foams, were calculated numerically by a PC code. Based on the superposition principle for linear systems, virtual complex loading of three PU foams’ cubes with densities of 34 kg/m^3^, 55 kg/m^3^, and 75 kg/m^3^ was considered. (1) Hydrostatic pressure σ_HP_ along with (2) shear were applied to the cubes’ faces parallel to the X_1_OX_2_, X_1_OX_3_, and X_2_OX_3_ planes:

σ_11_ = σ_22_ = σ_33_ = σ_HP_; τ_12_ = τ_21_ = τ, and τ_13_ = τ_31_ = τ_23_ = τ_32_ = τ′. (10)

Input data for the elastic constants and the stresses were provided to the PC code.

The experimental data identified some orthotropy of foams in the rectangular parallelepiped-shaped blocks: E_2_ > E_1_, ν_12_ > ν_21_, ν_13_ > ν_23_ (Tables S2–S5 of the Supplementary Materials). Therefore, constants linked to OX_1_ and OX_2_ directions were averaged:

E = ½(E_1_ + E_2_), ν = ½(ν_12_ + ν_21_), ν′ = ½(ν_31_ + ν_32_), ν″ = ½(ν_13_ + ν_23_), G′ = ½(G_13_ + G_23_), but G = G_12_, and E′ = E_3._ (11)

The modulus G was assumed to be equal to G_12_ (due to lacking experimental data for G_21_). The constants, calculated from Equations (11), are given in Table 3. The average anisotropy degree of the foams in blocks was estimated as A = E′/E [7] (Table 3).

Since the anisotropy degree A of foams in the three blocks is different, as seen in Table 3, the same-name moduli E_i_ and G_ij_ of the three blocks cannot be directly compared and the trendlines are not given in Figures S6 and S8 of the Supplementary Materials. It was shown in [10] that Poisson’s ratio depends on structural anisotropy and does not directly depend on density; therefore, the dependence of ν_12_, ν_13_, ν_21_, ν_23_, ν_31_, and ν_32_ on anisotropy degree is given in Figure S7 of the Supplementary Materials.

For an idealised, perfectly transtropic material, the density and degree of transtropy [10] are the same at all points of the block, so Equations (6) and (8) have to be satisfied exactly. In practice, the density and degree of transtropy vary from point to point in moulded blocks [3,4,7,8,10]. Therefore, it was necessary to evaluate the correspondence of the experimentally obtained elastic constants of PU foams (Tables S2–S5 of Supplementary Materials) to constants of a perfectly transtropic material. With this aim, the values of ratios f_1_ = ν″/E and f_2_ = ν′/E′ were calculated from Equations (6) and (11) with the experimental data from Tables S2–S4 of Supplementary Materials as the input. The standard uncertainties were estimated (in Supplementary Materials, section “Analysis of uncertainties”, points “1. Ratio f_1_” and “2. Ratio f_2_”) [28,29,30,31], and the ranges of f_1_ and f_2_ values were calculated. When necessary, expanded uncertainties U were estimated using the effective degrees of freedom and the coverage factor (Tables S7 and S8 of Supplementary Materials). The obtained ranges of f_1_ and f_2_ values were compared with each other. Then the values of modulus G were calculated from Equations (8) and (11) with the experimental data from Tables S2 and S3 of Supplementary Materials as the input. The standard uncertainties were estimated and the ranges of G values were calculated in Supplementary Materials, section “Analysis of uncertainties”, point “3. Modulus G”. The obtained ranges of G values were compared with the ranges of G values from direct experiments (Table S5 of Supplementary Materials).

The numerical results show that in the limits of the combined standard uncertainties u_c_(f_1_) and u_c_(f_2_), associated with the f_1_ and f_2_ estimates, there is no overlapping of the ranges of values of f_1_ and f_2_ (Table 4). Overlapping of the ranges of the values of f_1_ and f_2_ means that there exist such values of f_1_ and f_2_, for which Equation (6) is satisfied exactly: f_1_ = f_2_ = $\frac{\nu^{\prime }}{E}=\frac{\nu^{\prime \prime }}{E^{\prime }}$. The wider the overlap, the better the agreement of the experimental data set and that of a perfectly trantropic material.

In the limits of the expanded uncertainties U(f_1_) and U(f_2_), the range of f_1_ values overlaps with the range of f_2_ values for PU foams’ of average densities 34 kg/m^3^ and 75 kg/m^3^. At the average density 55 kg/m^3^, there is still no overlapping (Table 5). The two other variants of Equation (6) $\frac{E}{\nu^{\prime \prime }}=\frac{E^{\prime }}{\nu^{\prime }}$, and $\frac{\nu^{\prime \prime }}{\nu^{\prime }}=\frac{E}{E^{\prime }}$ provide similar results. It is concluded that Equation (6) is satisfied for the experimental data of the PU foam samples of average densities of 34 kg/m^3^ and 75 kg/m^3^ and is not satisfied for the data of foams with the average density of 55 kg/m^3^.

The numerical results show (Table 6) that already in the limits of standard uncertainties, the range of G values estimated from Equations (8) and (11) overlaps with the range of G values, determined from direct experiments (Table S5 of Supplementary Materials) for all the three considered average densities (34 kg/m^3^, 55 kg/m^3^, and 75 kg/m^3^). Therefore, there was no necessity to determine the range of G values corresponding to the expanded uncertainty U(G). It can be concluded that Equation (8) is satisfied for the experimental data of the PU foam samples of the average density 34 kg/m^3^, 55 kg/m^3^, and 75 kg/m^3^.

From the numerical results above, it was concluded that in the limits of expanded uncertainties ± U (≈95% of all random points, [28,29,30]), the experimentally obtained sets of elastic constants of PU foams correspond with the constants of a transtropic material for foams from the blocks of average densities 34 kg/m^3^ and 75 kg/m^3^. The sets of experimental elastic constants of PU foams from the block of average density 55 kg/m^3^ do not correspond with the constants of a transtropic material. These conclusions are valid only for the particular PU foam blocks produced under certain technological conditions, e.g., foams in a block with an average density of 55 kg/m^3^ produced under other technological conditions may well correspond to a transtropic material.

### 4.2. The Stresses of Complex Loading

The hydrostatic pressure has to ensure that response strains do not go beyond the elastic region of the uniaxial stress–strain curves. Therefore, the limit stresses σ_11lim_, σ_22lim_, and σ_33lim_ of the elastic region of experimental curves “σ_11_–ε_11_”, “σ_22_–ε_22_”, and “σ_33_–ε_33_” were determined and compared. Since σ_33lim_ > σ_11lim_, σ_22lim_ for the considered foams’ densities, σ_11lim_ and σ_22lim_ were selected for numerical calculations and the hydrostatic pressure was determined as σ_HP_ = ½(σ_11lim_ + σ_22lim_).

The shear stresses also have to ensure that the response strains do not go beyond the elastic region of shear stress–strain curves. The shear stress τ in the isotropy plane X_1_OX_2_ was determined as the limit stress τ_12lim_ of the elastic region of experimental curves “τ_12_–ε_12_”: τ = τ_12lim_. The shear stress τ′ in the X_1_OX_3_ and X_2_OX_3_ planes was determined as τ′ = ½(τ_13lim_ + τ_23lim_), where τ_13lim_ and τ_23lim_ are the limit stresses of the elastic region of experimental curves “τ_13_–ε_13_” and “τ_23_–ε_23_”.

The virtual stresses calculated for different densities of PU foams, are given in Table S6 of the Supplementary Materials. In order to stay in the elastic region at all the considered densities, the stresses σ_HP_ and τ, τ′, determined for the most light-weight PU foam’s (block No. 1, average density 34 kg/m^3^), were used in complex loading:

σ_HP_ = σ_HP_^1^ = ½(σ_11lim_^1^ + σ_22lim_^1^), τ = τ_12lim_^1^, and τ′ = ½(τ_13lim_^1^ + τ_23lim_^1^). (12)

The stresses, used in numerical calculations of response strains ε_ij_, are given in Table 7.

Then the strains ε_ij_ were calculated for the nine variants of independent constants at each average density of PU foams. The statistical characteristics (the average value, standard deviation, and coefficient of variation) were calculated for the set of nine values of each same-name strain ε_ij_:

(13) $$\varepsilon_{\mathrm{ijav}}\;=\;\frac{1}{9}\sum_{n=1}^{9}\varepsilon_{ij(n)},\;s_{\mathrm{ij}}\;=\;\sqrt{\frac{\sum_{n=1}^{9}{\left[\varepsilon_{ij\left(n\right)}-\varepsilon_{ijav}\right]}^{2}}{8}}\;\mathrm{and}\;v_{\mathrm{ij}}\;=\;\frac{s_{ij}}{\varepsilon_{ijav}};$$

where i, j = 1, 2, 3 and the ordinal number “n” of a variant is a non-tensorial index n = 1, 2, …, 9. The relative difference between the average strain ε_ijav_ and the same-name strain ε_ij(n)_ for the n-th variant of independent constants was estimated as

(14) $$R_{\mathrm{ij}(n)}=\frac{\varepsilon_{ijav}-\varepsilon_{ij(n)}}{\varepsilon_{ijav}},\;\mathrm{where}\;n=1,\;2,\;\ldots ,\;9.$$

The set of average values of strains ε_11av_, ε_22av_, ε_33av_, ε_23av_, ε_31av_, and ε_12av_ characterises, in a certain sense, the set of strains of perfectly transtropic PU foams. Therefore, the value of summary relative difference between each of the six average strains ε_kav_ and each of the six same-name strains ε_k(n)_ was calculated for the nine variants of independent constants:

(15) $$R_{\left(n\right)}\;=\;\sum_{k=1}^{6}|R_{k(n)}|=\sum_{k=1}^{6}\frac{|(\varepsilon_{kav}-\varepsilon_{k(n)})|}{\varepsilon_{kav.}}$$

where n = 1, 2, …, 9 and k = 1 at ij = 11, k = 2 at ij = 22, k = 3 at ij = 33, k = 4 at ij = 23, k = 5 at ij = 31, and k = 6 at ij = 12. The variant of independent constants, which provided the smallest summary relative difference R_(n)_, was considered to have the best conformity with the constants of perfectly transtropic PU foams.

## 5. Results and Discussion

The numerical results for response strains ε_ij_ at the nine variants of independent constants are given in Table S9 of the Supplementary Materials. It can be seen that the highest strains correspond to the PU foams of the lowest average density 34 kg/m^3^. At higher average densities of 55 kg/m^3^ and 75 kg/m^3^, the strains are smaller both in hydrostatic pressure and shear due to higher elastic moduli. All strains remain in the linear regions of the stress–strain diagrams. The same-name strains are different for the nine variants of independent constants; the coefficient of variations for ε_11_, ε_22_, ε_23_, ε_31_, and ε_12_ is within a range 0% ≤ v ≤ 21%. The high values of v for the strain ε_33_ (111%, 171%, and 33%) are caused by the comparatively small absolute values of ε_33_ at different variants when any deviation of an input constant from the perfect value causes a comparatively high deviation from the average in the value of ε_33_.

The average strains in the plane of isotropy X_1_OX_2_ are equal and considerably higher than those along the rise direction OX_3_: ε_11av_ = ε_22av_ and |ε_11av_|, |ε_22av_| >> |ε_33av_| (Tables S9 and S10 of the Supplementary Materials). The fifth variant of constants (G_13_, G_12_, E_3_, ν_12_, and ν_13_) provides the best correspondence with constants of a transtropic material for foams of average density 34 kg/m^3^ and the fourth variant (G_13_, G_12_, E_3_, ν_12_, and ν_31_) for foams with an average density of 75 kg/m^3^ because these variants ensure the smallest summary relative differences R_(5)_ = 2.640 and R_(4)_ = 0.273 (Table S10 of the Supplementary Materials). The average strains of the foams in the block of average density 55 kg/m^3^ (which does not correspond to a perfectly monotropic material), have the biggest coefficients of variations, as shown in Table 8.

When a material is perfectly transtropic, in the limits of uncertainties, all variants of the experimentally determined independent elastic constants have to provide equal numerical results for same-name strain components ε_ij_ of the system of Equations (5). However, in practice, the distribution of density and anisotropy degree in the moulded rectangular and cylindrical blocks of free-rise PU foams is non-uniform, as indicated by the density gradients and convex surface of rise [3], e.g., a density gradient dρ/dx = −10 kg/m^3^/5 cm from edge to centre of a rectangular free-rise block of average density 74 kg/m^3^ is reported in [27]. Then the testing samples have different densities and degrees of transtropy depending on location in the block (Tables S2–S5 of the Supplementary Materials). As a result, the set of the measured elastic constants does not fully conform to the set of constants of transtropic material and the strain components are different at different variants.

The non-uniform distribution of density and anisotropy degree throughout a PU foam’s block is mainly due to technological conditions (kind and rate of foaming, size and temperature of the mould, ambient environment, etc.) [2,3,4,10] and, as such, it is present in PU foam blocks of any density. The conformity of the experimental constants to those of a perfectly transtropic material can be improved by achieving a sufficient number of large blocks produced under similar technological conditions to ensure making of the testing samples from compact volumes of similar locations in the blocks. The distribution of density and anisotropy degree in the blocks should be studied prior to any other measurements.

## 6. Conclusions

(1) Symmetry elements of rigid PU foams were considered in connection with characteristics of production moulds and technologies. The variants of independent elastic constants were determined for orthotropic, orthotropic with a rotational symmetry, and isotropic PU foams.
(2) Nine variants of independent elastic constants were identified for transtropic PU foams and corresponding equations of the generalised Hooke’s law were derived for the components of response strain.
(3) Correspondence of the experimentally determined elastic constants of rigid PU foams with constants of perfectly transtropic material was assessed, based on analysis of satisfying of the transtropy equations and of measurement uncertainties.
(4) The variants of independent constants were outlined, providing the best conformity with the set of average strains, which characterises in certain meaning the set of strains of perfectly transtropic PU foams. The non-uniform distribution of density and anisotropy degree in the PU foam blocks are suggested as the main reason for the experimental constants not fully corresponding to those of a transtropic material.
(5) The variant of independent elastic constants, which is the most appropriate for experimental determination or mathematical modelling, has to be selected in practice. All nine variants comprise the shear modulus G_13_, therefore the height of the moulded PU foam blocks has to be sufficient for such a length of shear samples, which ensures prevalence of shear over bending.
(6) No limitations specific to PU foams were implemented; therefore, the variants of independent elastic constants are valid for other materials with symmetry elements like other plastic foams, fibreglass–plastic composites, veneer, wood, etc. Further research may be directed towards determining variants of independent elastic constants using the elastic potential.

## Figures and Tables

**Table 1:** Variants of independent constants of orthotropic PU foams; n is the ordinal number of a variant.

n	Variant	n	Variant
1	G_23_, G_13_, G_12_, E_1_, E_2_, E_3_, ν_21_, ν_31_, ν_32_	5	G_23_, G_13_, G_12_, E_1_, E_2_, E_3_, ν_12_, ν_31_, ν_32_
2	G_23_, G_13_, G_12_, E_1_, E_2_, E_3_, ν_21_, ν_31_, ν_23_	6	G_23_, G_13_, G_12_, E_1_, E_2_, E_3_, ν_12_, ν_31_, ν_23_
3	G_23_, G_13_, G_12_, E_1_, E_2_, E_3_, ν_21_, ν_13_, ν_32_	7	G_23_, G_13_, G_12_, E_1_, E_2_, E_3_, ν_12_, ν_13_, ν_32_
4	G_23_, G_13_, G_12_, E_1_, E_2_, E_3_, ν_21_, ν_13_, ν_23_	8	G_23_, G_13_, G_12_, E_1_, E_2_, E_3_, ν_12_, ν_13_, ν_23_

**Table 2:** Variants of independent elastic constants of transtropic PU foams; n is the ordinal number of the variants.

n	Variant	n	Variant	n	Variant
1	G_13_, E_1_, E_3_, ν_12_, ν_31_	4	G_13_, G_12_, E_3_, ν_12_, ν_31_	7	G_13_, G_12_, E_1_, E_3_, ν_31_
2	G_13_, E_1_, E_3_, ν_12_, ν_13_	5	G_13_, G_12_, E_3_, ν_12_, ν_13_	8	G_13_, G_12_, E_1_, E_3_, ν_13_
3	G_13_, E_1_, ν_12_, ν_31_, ν_13_	6	G_13_, G_12_, ν_12_, ν_31_, ν_13_	9	G_13_, G_12_, E_1_, ν_31_, ν_13_

**Table 3:** The averaged experimental data of PU foams.

Average Density ρ_av_; kg/m^3^	E; MPa	ν	E′; MPa	ν′	ν″	G; MPa	G′; MPa	A
34	4.3	0.29	10.4	0.48	0.23	1.8	2.6	2.4
55	11.3	0.36	19.4	0.51	0.22	3.9	5.0	1.7
75	19.7	0.31	28.7	0.41	0.24	7.1	9.0	1.5

**Table 4:** The ranges of values of the functions f_1_ and f_2_ at the combined standard uncertainties u_c_.

Average Density ρ_av_; kg/m^3^	f_1_	f_2_	u_c_(f_1_)	u_c_(f_2_)	Range of f_1_ Values	Range of f_2_ Values
34	0.053	0.046	0.0022	0.0026	0.051 ≤ f_1_ ≤ 0.055	0.044 ≤ f_2_ ≤ 0.049
55	0.019	0.026	0.0005	0.0009	0.019 ≤ f_1_ ≤ 0.020	0.025 ≤ f_2_ ≤ 0.027
75	0.012	0.014	0.0006	0.0006	0.011 ≤ f_1_ ≤ 0.013	0.014 ≤ f_2_ ≤ 0.015

**Table 5:** The ranges of values of the functions f_1_ and f_2_ at the expanded uncertainties U.

Average Density ρ_av_; kg/m^3^	f_1_	f_2_	U(f_1_)	U(f_2_)	Range of f_1_ Values	Range of f_2_ Values
34	0.053	0.046	0.0052	0.0069	0.048 ≤ f_1_ ≤ 0.058	0.039 ≤ f_2_ ≤ 0.053
55	0.019	0.026	0.0012	0.0025	0.018 ≤ f_1_ ≤ 0.021	0.024 ≤ f_2_ ≤ 0.028
75	0.012	0.014	0.0014	0.0017	0.011 ≤ f_1_ ≤ 0.013	0.013 ≤ f_2_ ≤ 0.016

**Table 6:** The range of values of modulus G at the combined standard uncertainty u_c_.

Average Density ρ_av_; kg/m^3^	Equations (8) and (11)			Direct Experimental Data		
	G; MPa	u_c_(G)	Range of G Values	G; MPa	u_c_(G)	Range of G Values
34	1.7	0.05	1.6 ≤ G ≤ 1.7	1.8	0.25	1.55 ≤ G ≤ 2.05
55	4.2	0.09	4.1 ≤ G ≤ 4.3	3.9	0.35	3.55 ≤ G ≤ 4.25
75	7.5	0.26	7.3 ≤ G ≤ 7.8	7.1	0.30	6.80 ≤ G ≤ 7.40

**Table 7:** The stresses of virtual complex loading.

Average Density ρ_av_; kg/m^3^	Hydrostatic Pressure σ_HP_; MPa	Shear Stress	
		τ′; MPa	τ; MPa
34, 55 and 75	−0.045	0.042	0.030

**Table 8:** The average strains of rigid PU foams; v is the coefficient of variations.

Average Strain	ρ = 34 kg/m^3^		ρ = 55 kg/m^3^		ρ = 75 kg/m^3^	
	ε_ijav_	v; %	ε_ijav_	v; %	ε_ijav_	v; %
ε_11av_	−0.0054	14	−0.0015	21	−0.0010	13
ε_22av_	−0.0054	13	−0.0015	17	−0.0010	11
ε_33av_	−0.0001	111	−0.0002	171	−0.0003	33
ε_23av_	0.0082	0	0.0043	0	0.0024	0
ε_31av_	0.0082	0	0.0043	0	0.0024	0
ε_12av_	0.0086	4	0.0038	4	0.0021	3

## Data Availability

The original contributions presented in this study are included in the article/supplementary material. Further inquiries can be directed to the corresponding authors.

## Supplementary Materials

The following supporting information can be downloaded at: https://www.mdpi.com/article/10.3390/polym17172431/s1, Table S1. Variants of independent elastic constants of a transtropic material, Table S2. Mechanical properties of PU foams in compression parallel to axis OX_1_ (the experimental standard deviation ± s and coefficient of variations v, in %), Table S3. Mechanical properties of PU foams in compression parallel to axis OX_2_ (the experimental standard deviation ± s and coefficient of variations v, in %), Table S4. Mechanical properties of PU foams in compression parallel to axis OX_3_ (the experimental standard deviation ± s and coefficient of variations v, in %), Table S5. Mechanical properties of PU foams in shear (the experimental standard deviation ± s and coefficient of variations v, in %), Table S6. The calculated virtual stresses at different densities of PU foams, Table S7. The effective degrees of freedom and coverage factor, Table S8. The effective degrees of freedom and coverage factor, Table S9. The strains ε_ij_ at nine variants of independent constants of transtropic PU foams (v is the coefficient of variation, in %), and Table S10. The relative difference R_k(n)_ between the strains εk(n) and the averaged strains ε_kav_, as well as the summary relative difference R_(n)_ for the six strains. Figure S1. PU foams’ block ( 500 mm × 600 mm × 200 mm); 1 – a parallelepiped of compression samples (150 mm × 200 mm × 100 mm) and 2 – a parallelpepiped of shear samples (100 mm × 122 mm × 100 mm); OX_1_, OX_2_ and OX_3_—main directions of density gradients, Figure S2. The parallelepiped 1 of samples for compression parallel to axis 1) OX_1_ (E1a, E1b, E1c and E1d), 2) OX_2_ (E2a, E2b, E2c and E2d) and 3) OX_3_ (E3a, E3b, E3c and E3d), Figure S3. The parallelepiped of samples for shear in plane 1) X_1_OX_2_ (G12a, G12b, G12c, and G12d), 2) X_3_OX_2_ (G32a, G32b, G32c and G32d), and 3) X_3_OX_1_ (G31a, G31b, G31c, and G31d), Figure S4. Stress – strain curves of PU foams of average density 55 kg/m^3^ in compression parallel to axis 1) OX_1_, 2) OX_2_, and 3) OX_3_; “- - - - -“ a tangent to the longitudinal stress - strain curve, “− − −” a straight for identification of the elastic region at the crossing point with the tangent, Figure S5. Stress – strain curves of PU foams of average density 55 kg/m^3^ in shear in planes 1) X_1_OX_2_, 2) X_1_OX_3_, and 3) X_2_OX_3_. “- - - - -“ a tangent to the longitudinal stress - strain curve, “− − −” a straight for identification of the elastic region at the crossing point with the tangent, Figure S6. Moduli E_1_, E_2_ and E_3_ of PU foams in the bocks of average density 34 kg/m^3^, 55 kg/m^3^ and 75 kg/m^3^, Figure S7. Poisson’s ratios ν_12_, ν_13_, ν_21_, ν_23_, ν_31_ and ν_32_ of PU foams in dependence of average anisotropy degree A (ν = 0.33 is Poisson’s ratio of isotropic PU foams), and Figure S8. Shear moduli G_12_, G_13_ and G_23_ of PU foams in the bocks of average density 34 kg/m^3^, 55 kg/m^3^ and 75 kg/m^3^.
