# Supplementary material for: Investigation of the Variants of Independent Elastic Constants of Rigid Polyurethane Foams with Symmetry Elements
Source: Polymers (Basel). 2025 Sep 8;17(17):2431. doi: 10.3390/polym17172431 (PMC12431319; doi:10.3390/polym17172431)
Supplement: Supplementary file 1 [file polymers-17-02431-s001.zip › polymers-3811740-Supplementary Materials-final.pdf]

Supplementary Materials

Table S1. Variants of independent elastic constants of a transtropic material.

Elastic constants				Independent elastic constants	Number n
1	2	3	4	5	6
G′	E, ν	E, E′, ν′	G′, E, E′, ν, ν′	G_{13}, E_1, E_3, ν_{12}, ν_{31}	1
		E, E′, ν′′	G′, E, E′, ν, ν′′	G_{13}, E_1, E_3, ν_{12}, ν_{13}	2
		E, ν′, ν′′	G′, E, ν, ν′, ν′′	G_{13}, E_1, ν_{12}, ν_{31}, ν_{13}	3
		E′, ν′, ν′′	G′, E, E′, ν, ν′, ν′′ (6)		
			Expressed: E′, ν′, ν′′		
			G′, E, ν, ν′, ν′′	G_{13}, E_1, ν_{12}, ν_{31}, ν_{13}	Repeat 2
			G′, E, E′, ν, ν′′	G_{13}, E_1, E_3, ν_{12}, ν_{13}	Repeat 3
			G′, E, E′, ν, ν′	G_{13}, E_1, E_3, ν_{12}, ν_{31}	Repeat 1
	G, ν	E, E′, ν′	G′, G, E, E′, ν, ν′ (6)		
			Expressed: E, E′, ν′		
			G′, G, E′, ν, ν′	G_{13}, G_{12}, E_3, ν_{12}, ν_{31}	4
			G′, G, E, ν, ν′, ν′′ (6)		
			G′, G, E, E′, ν, ν′′ (6)		
		E, E′, ν′′	G′, G, E, E′, ν, ν′′ (6)		
			Expressed: E, E′, ν′′		
			G′, G, E′, ν, ν′′	G_{13}, G_{12}, E_3, ν_{12}, ν_{13}	5
			G′, G, E, ν, ν′, ν′′ (6)		
			G′, G, E, E′, ν, ν′ (6)		
		E, ν′, ν′′	G′, G, E, ν′, ν, ν′′ (6)		
			Expressed: E, ν′, ν′′		
			G′, G, ν′, ν, ν′′	G_{13}, G_{12}, ν_{12}, ν_{31}, ν_{13}	6
			G′, G, E, E′, ν, ν′′ (6)		
			G′, G, E, ν′, ν, E′ (6)		
		E′, ν′, ν′′	G′, G, ν, E′, ν′, ν′′ (6)		
			Expressed: E′, ν′, ν′′		
			G′, G, ν, E, ν′, ν′′ (6)	-	-
			G′, G, ν, E′, E, ν′′ (6)	-	-
			G′, G, ν, E′, E, ν′ (6)	-	-
	G, E	E, E′, ν′	G′, G, E, E′, ν′	G_{13}, G_{12}, E_1, E_3, ν_{31}	7
		E, E′, ν′′	G′, G, E, E′, ν′′	G_{13}, G_{12}, E_1, E_3, ν_{13}	8
		E, ν′, ν′′	G′, G, E, ν′, ν′′	G_{13}, G_{12}, E_1, ν_{31}, ν_{13}	9
		E′, ν′, ν′′	G′, G, E, E′, ν′, ν′′ (6)		
			Expressed E′, ν′, ν′′		
			G′, G, E, E′, ν′′	G_{13}, G_{12}, E_1, E_3, ν_{13}	Repeat 8
			G′, G, E, E′, ν′	G_{13}, G_{12}, E_1, E_3, ν_{31}	Repeat 7
			G′, G, E, ν′, ν′′	G_{13}, G_{12}, E_1, ν_{31}, ν_{13}	Repeat 9

Variant 1. G′, E′, E, ν, ν′

$$\varepsilon_{11} = \frac{1}{E}(\sigma_{11} - \nu\sigma_{22}) - \frac{\nu'}{E'}\sigma_{33},$$

$$\varepsilon_{22} = \frac{1}{E}(-\nu\sigma_{11} + \sigma_{22}) - \frac{\nu'}{E'}\sigma_{33},$$

Variant 6. G′, G, ν, ν′, ν′′

$$\varepsilon_{11} = \frac{1}{2G(1+\nu)}(\sigma_{11} - \nu\sigma_{22}) - \frac{\nu''}{2G(1+\nu)}\sigma_{33},$$

$$\varepsilon_{22} = \frac{1}{2G(1+\nu)}(-\nu\sigma_{11} + \sigma_{22}) - \frac{\nu''}{2G(1+\nu)}\sigma_{33},$$

$$\varepsilon_{33} = -\frac{\nu'}{E'}(\sigma_{11} + \sigma_{22}) + \frac{1}{E'}\sigma_{33},$$

$$\varepsilon_{23} = \frac{1}{2G'}\tau_{23},$$

(S1)

$$\varepsilon_{31} = \frac{1}{2G'}\tau_{31} \text{ and}$$

$$\varepsilon_{12} = \frac{1+\nu}{E}\tau_{12}.$$

Variant 2. G′, E, E′, ν, ν′′

$$\varepsilon_{11} = \frac{1}{E}(\sigma_{11} - \nu\sigma_{22}) - \frac{\nu''}{E}\sigma_{33},$$

$$\varepsilon_{22} = \frac{1}{E}(-\nu\sigma_{11} + \sigma_{22}) - \frac{\nu''}{E}\sigma_{33},$$

$$\varepsilon_{33} = -\frac{\nu''}{E}(\sigma_{11} + \sigma_{22}) + \frac{1}{E'}\sigma_{33},$$

$$\varepsilon_{23} = \frac{1}{2G'}\tau_{23},$$

(S2)

$$\varepsilon_{31} = \frac{1}{2G'}\tau_{31} \text{ and}$$

$$\varepsilon_{12} = \frac{1+\nu}{E}\tau_{12}.$$

Variant 3. G′, E, ν, ν′, ν′′

$$\varepsilon_{11} = \frac{1}{E}(\sigma_{11} - \nu\sigma_{22}) - \frac{\nu''}{E}\sigma_{33},$$

$$\varepsilon_{22} = \frac{1}{E}(-\nu\sigma_{11} + \sigma_{22}) - \frac{\nu''}{E}\sigma_{33},$$

$$\varepsilon_{33} = -\frac{\nu''}{E}(\sigma_{11} + \sigma_{22}) + \frac{\nu''}{\nu' E}\sigma_{33},$$

$$\varepsilon_{23} = \frac{1}{2G'}\tau_{23},$$

(S3)

$$\varepsilon_{31} = \frac{1}{2G'}\tau_{31} \text{ and}$$

$$\varepsilon_{12} = \frac{1+\nu}{E}\tau_{12}.$$

Variant 4. G′, G, E′, ν, ν′

$$\varepsilon_{11} = \frac{1}{2G(1+\nu)}(\sigma_{11} - \nu\sigma_{22}) - \frac{\nu'}{E'}\sigma_{33},$$

$$\varepsilon_{22} = \frac{1}{2G(1+\nu)}(-\nu\sigma_{11} + \sigma_{22}) - \frac{\nu'}{E'}\sigma_{33},$$

$$\varepsilon_{33} = -\frac{\nu'}{E'}(\sigma_{11} + \sigma_{22}) + \frac{1}{E'}\sigma_{33},$$

$$\varepsilon_{23} = \frac{1}{2G'}\tau_{23},$$

(S4)

$$\varepsilon_{31} = \frac{1}{2G'}\tau_{31} \text{ and}$$

$$\varepsilon_{12} = \frac{1}{2G}\tau_{12}.$$

Variant 5. G′, G, E′, ν, ν′′

$$\varepsilon_{11} = \frac{1}{2G(1+\nu)}(\sigma_{11} - \nu\sigma_{22}) - \frac{\nu''}{E}\sigma_{33},$$

$$\varepsilon_{22} = \frac{1}{2G(1+\nu)}(-\nu\sigma_{11} + \sigma_{22}) - \frac{\nu''}{E}\sigma_{33},$$

$$\varepsilon_{33} = -\frac{\nu''}{2G(1+\nu)}(\sigma_{11} + \sigma_{22}) + \frac{\nu''}{2G\nu'(1+\nu)}\sigma_{33},$$

$$\varepsilon_{23} = \frac{1}{2G'}\tau_{23},$$

(S6)

$$\varepsilon_{31} = \frac{1}{2G'}\tau_{31} \text{ and}$$

$$\varepsilon_{12} = \frac{1}{2G}\tau_{12}.$$

Variant 7. G′, G, E′, E, ν′

$$\varepsilon_{11} = \frac{1}{E}\left[\sigma_{11} + \frac{(2G-E)}{2G}\sigma_{22}\right] - \frac{\nu'}{E'}\sigma_{33},$$

$$\varepsilon_{22} = \frac{1}{E}\left[\frac{(2G-E)}{2G}\sigma_{11} + \sigma_{22}\right] - \frac{\nu'}{E'}\sigma_{33},$$

$$\varepsilon_{33} = -\frac{\nu'}{E'}(\sigma_{11} + \sigma_{22}) + \frac{1}{E'}\sigma_{33},$$

$$\varepsilon_{23} = \frac{1}{2G'}\tau_{23},$$

(S7)

$$\varepsilon_{31} = \frac{1}{2G'}\tau_{31} \text{ and}$$

$$\varepsilon_{12} = \frac{1}{2G}\tau_{12}.$$

Variant 8. G′, G, E, E′, ν′′

$$\varepsilon_{11} = \frac{1}{E}\left[\sigma_{11} + \frac{(2G-E)}{2G}\sigma_{22}\right] - \frac{\nu''}{E}\sigma_{33},$$

$$\varepsilon_{22} = \frac{1}{E}\left[\frac{(2G-E)}{2G}\sigma_{11} + \sigma_{22}\right] - \frac{\nu''}{E}\sigma_{33},$$

$$\varepsilon_{33} = -\frac{\nu''}{E}(\sigma_{11} + \sigma_{22}) + \frac{1}{E'}\sigma_{33},$$

$$\varepsilon_{23} = \frac{1}{2G'}\tau_{23},$$

(S8)

$$\varepsilon_{31} = \frac{1}{2G'}\tau_{31} \text{ and}$$

$$\varepsilon_{12} = \frac{1}{2G}\tau_{12}.$$

Variant 9. G′, G, E, ν′, ν′′

$$\varepsilon_{11} = \frac{1}{E}\left[\sigma_{11} + \frac{(2G-E)}{2G}\sigma_{22}\right] - \frac{\nu''}{E}\sigma_{33},$$

$$\varepsilon_{22} = \frac{1}{E}\left[\frac{(2G-E)}{2G}\sigma_{11} + \sigma_{22}\right] - \frac{\nu''}{E}\sigma_{33},$$

$$\varepsilon_{33} = -\frac{\nu''}{E}(\sigma_{11} + \sigma_{22}) + \frac{\nu''}{\nu' E}\sigma_{33},$$

$$\varepsilon_{23} = \frac{1}{2G'}\tau_{23},$$

(S9)

$$\varepsilon_{31} = \frac{1}{2G'}\tau_{31} \text{ and}$$

$$\varepsilon_{12} = \frac{1}{2G}\tau_{12}.$$

$\varepsilon_{33} = -\frac{\nu''}{2G(1+\nu)}(\sigma_{11} + \sigma_{22}) + \frac{1}{E'}\sigma_{33},$

$\varepsilon_{23} = \frac{1}{2G'}\tau_{23},$

(S5)

$\varepsilon_{31} = \frac{1}{2G'}\tau_{31}$ and

$\varepsilon_{12} = \frac{1}{2G}\tau_{12}.$

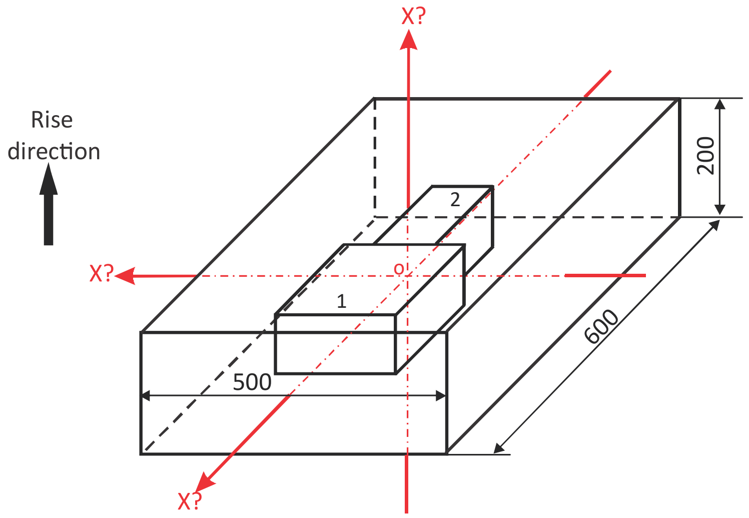

Figure S1. PU foams' block (500 mm × 600 mm × 200 mm); 1 – a parallelepiped of compression samples (150 mm × 200 mm × 100 mm) and 2 – a parallelpepiped of shear samples (100 mm × 122 mm × 100 mm); OX_1, OX_2 and OX_3 – main directions of density gradients.

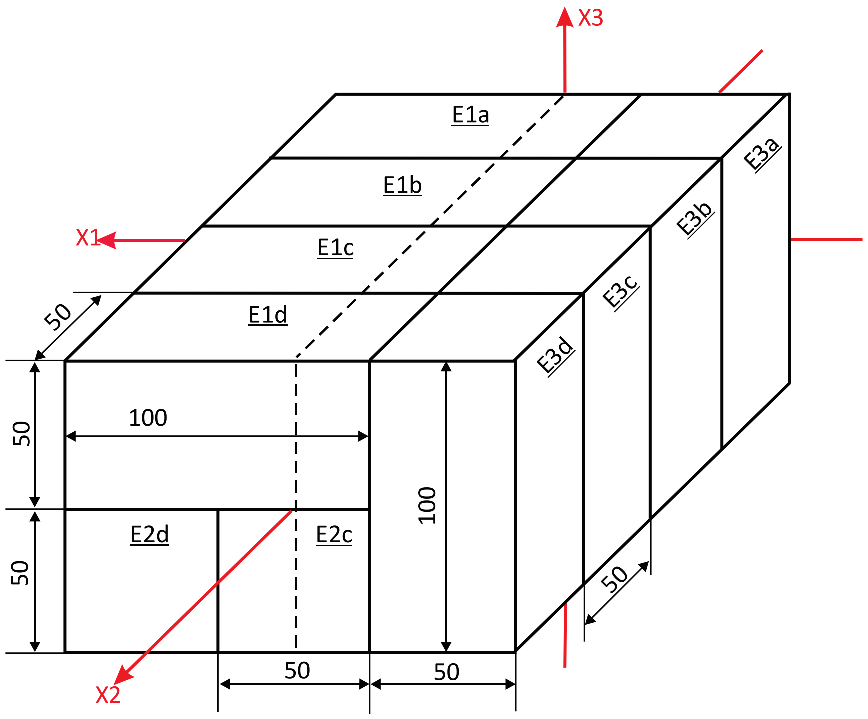

Figure S2. The parallelepiped 1 of samples for compression parallel to axis 1) OX_1 (E1a, E1b, E1c and E1d), 2) OX_2 (E2a, E2b, E2c and E2d) and 3) OX_3 (E3a, E3b, E3c and E3d).

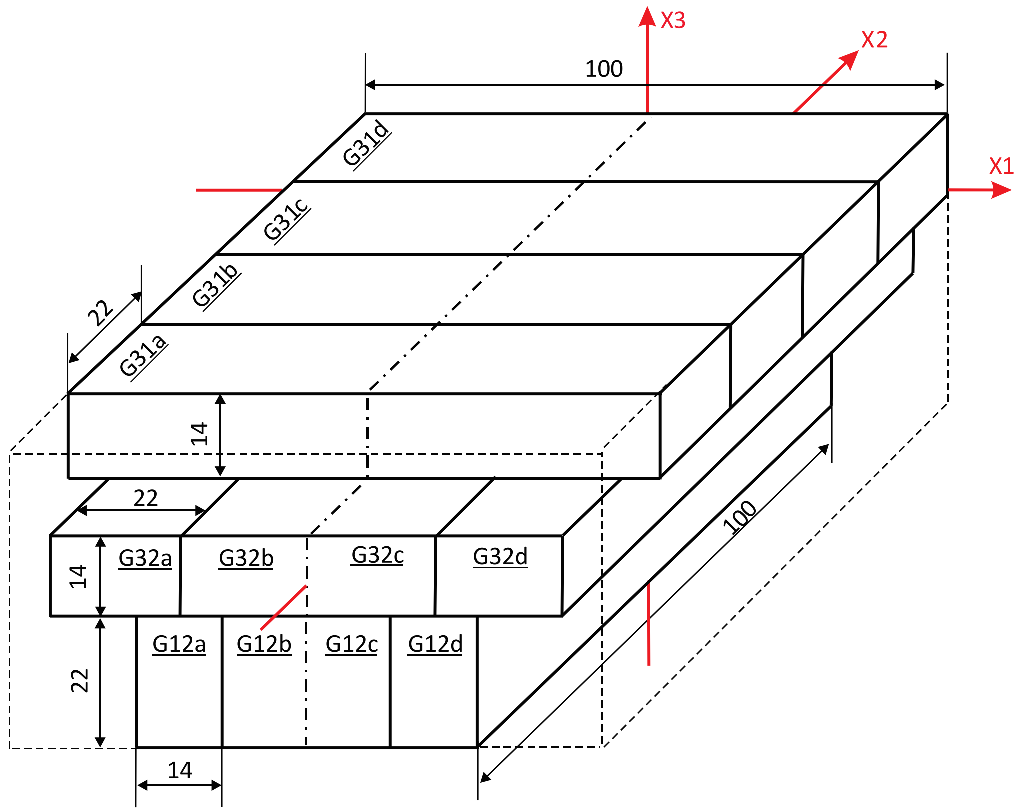

Figure S3. The parallelepiped of samples for shear in plane 1) X_1OX_2 (G12a, G12b, G12c, and G12d), 2) X_3OX_2 (G32a, G32b, G32c and G32d), and 3) X_3OX_1 (G31a, G31b, G31c, and G31d).

1)

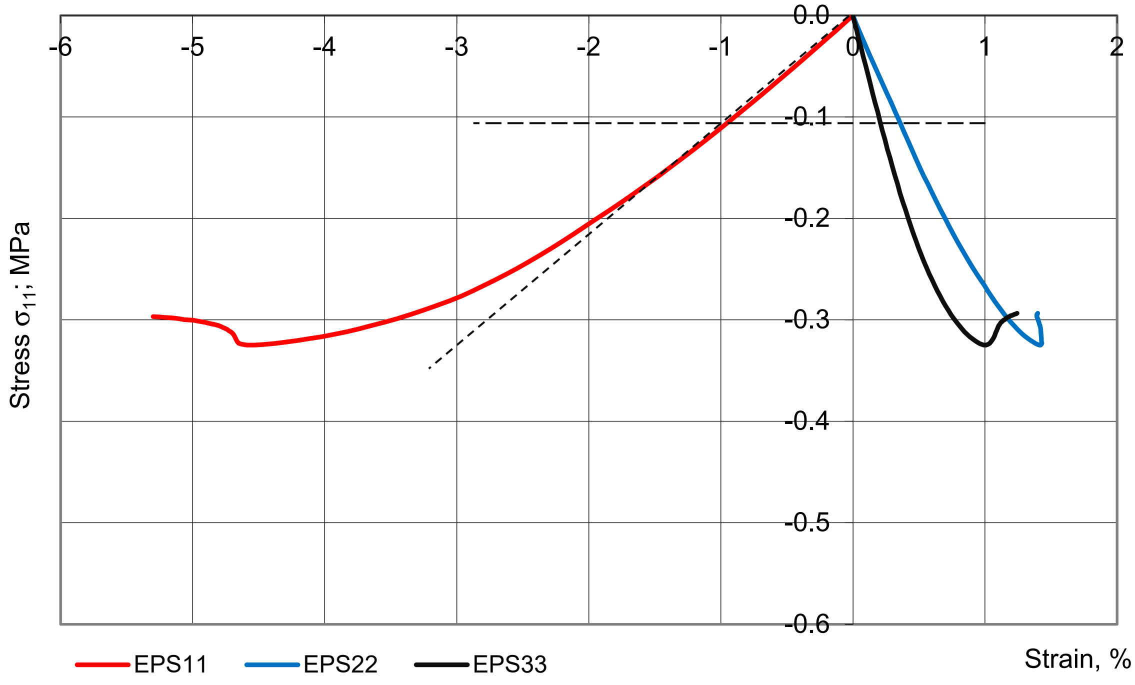

2)

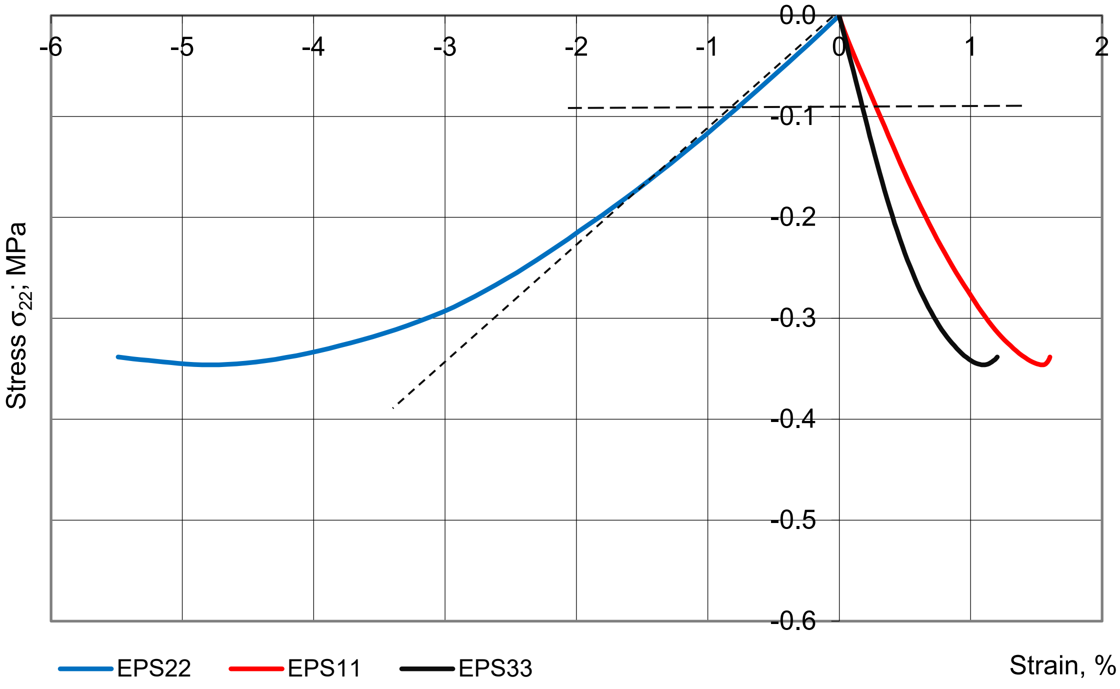

3)

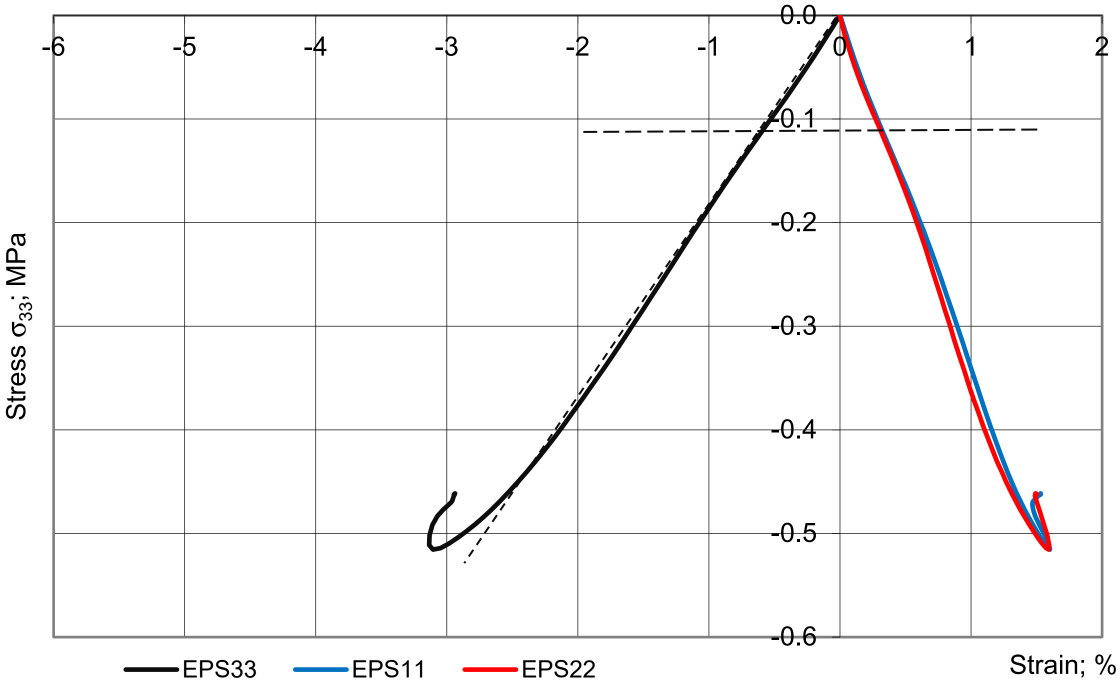

Figure S4. Stress – strain curves of PU foams of average density 55 kg/m^3 in compression parallel to axis 1) OX_1, 2) OX_2, and 3) OX_3; "- - - - -" a tangent to the longitudinal stress - strain curve, "– – –" a straight for identification of the elastic region at the crossing point with the tangent.

1)

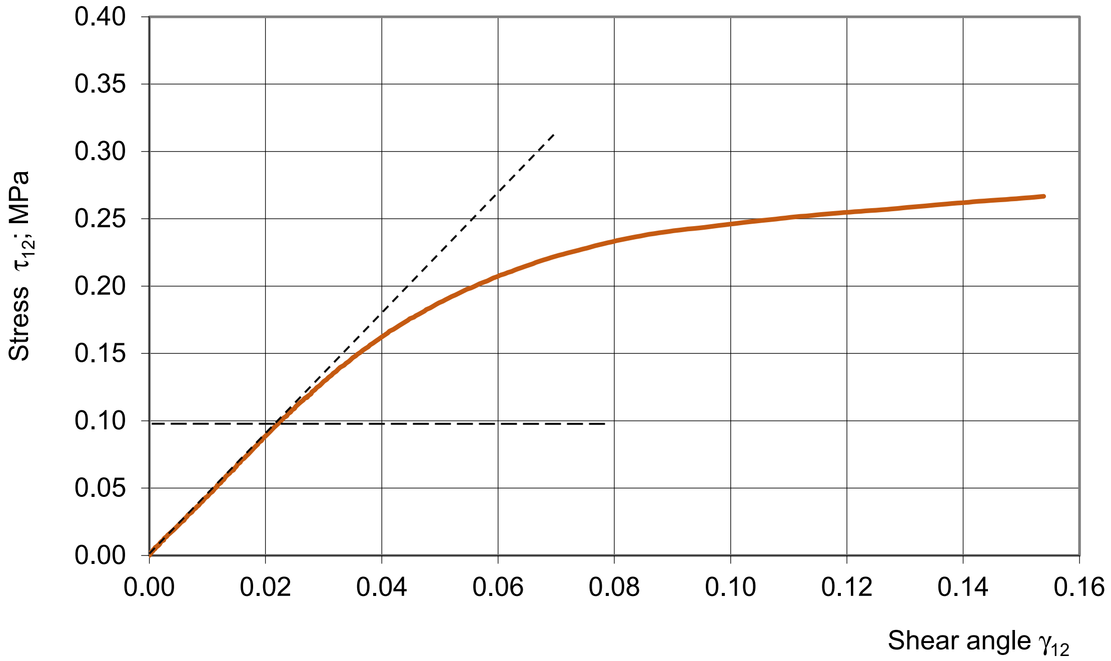
0.40
0.35
0.30
0.25
0.20
0.15
0.10
0.05
0.00
Stress τ_{12}; MPa
0.00
0.02
0.04
0.06
0.08
0.10
0.12
0.14
0.16
Shear angle γ_{12}

2)

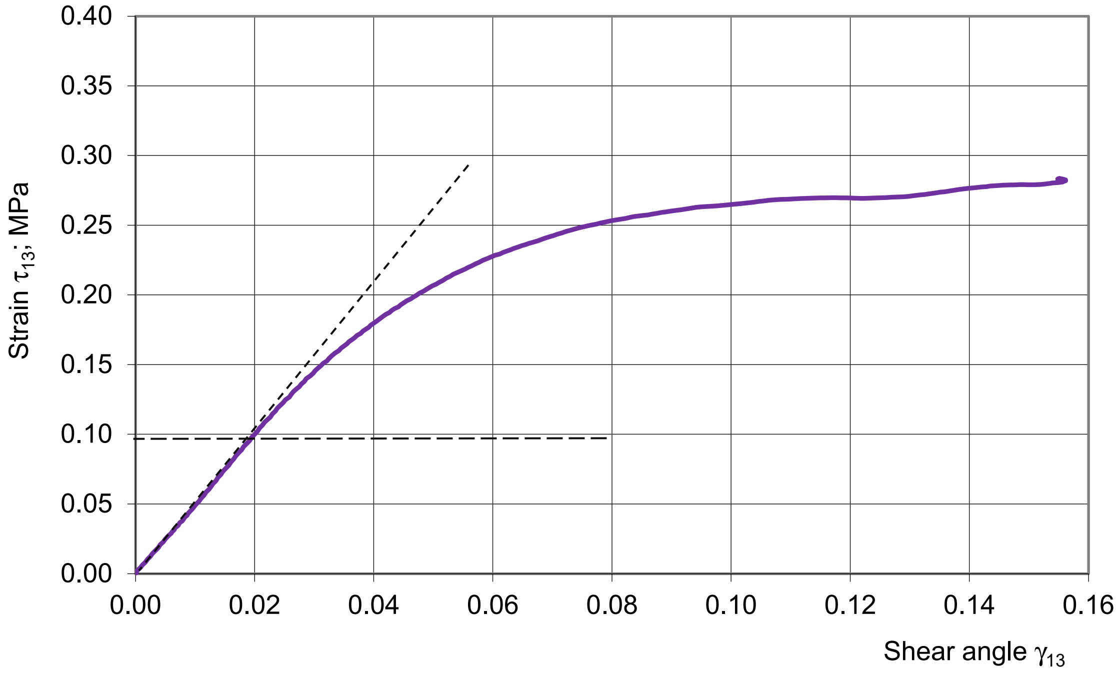
0.40
0.35
0.30
0.25
0.20
0.15
0.10
0.05
0.00
Strain τ_{13}; MPa
0.00
0.02
0.04
0.06
0.08
0.10
0.12
0.14
0.16
Shear angle γ_{13}

3)

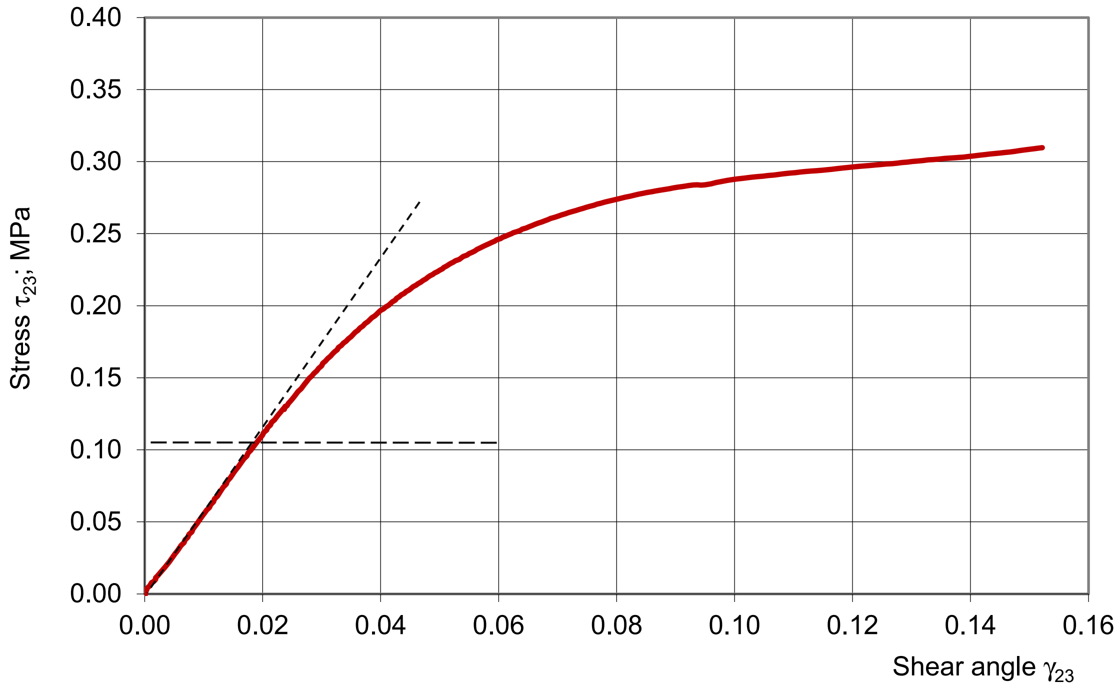

Figure S5. Stress – strain curves of PU foams of average density 55 kg/m^3 in shear in planes 1) X_1OX_2, 2) X_1OX_3, and 3) X_2OX_3. "- - - - -" a tangent to the longitudinal stress - strain curve, "– – –" a straight for identification of the elastic region at the crossing point with the tangent.

Table S2. Mechanical properties of PU foams in compression parallel to axis OX_1 (the experimental standard deviation ± s and coefficient of variations v, in %).

Property	Number of a block					
	1		2		3	
Density ρ; kg/m^3	33	± 0.1 (0 %)	56	± 0.3 (0 %)	76	± 0.3 (0 %)
Modulus E_1; MPa	3.7	± 0.4 (11 %)	9.8	± 0.2 (2 %)	17.8	± 1.6 (9 %)
Poisson's ratio ν_{12}	0.27	± 0.02 (7 %)	0.33	± 0.01 (3 %)	0.30	± 0.03 (10 %)
Poisson's ratio ν_{13}	0.21	± 0.01 (5 %)	0.23	± 0.01 (4 %)	0.22	± 0.03 (14 %)
Stress σ_{11lim}; MPa	– 0.04	± 0.00 (0 %)	– 0.08	± 0.02 (25 %)	– 0.13	± 0.02 (15 %)
Strain ε_{11lim}	– 0.011	± 0.001 9 %)	– 0.008	± 0.002 (25 %)	– 0.007	± 0.002 (29%)

Table S3. Mechanical properties of PU foams in compression parallel to axis OX_2 (the experimental standard deviation ± s and coefficient of variations v, in %).

Property	Number of a block					
	1		2		3	
Density ρ; kg/m^3	33	± 0.2 (1 %)	54	± 0.2 (0 %)	77	± 0.7 (1 %)
Modulus E_2; MPa	4.8	± 0.5 (10 %)	12.9	± 0.9 (7 %)	21.5	± 2.6 (12 %)
Poisson´s ratio ν_{21}	0.30	± 0.02 (7 %)	0.38	± 0.02 (5 %)	0.31	± 0.02 (6 %)
Poisson´s ratio ν_{23}	0.24	± 0.01 (4 %)	0.21	± 0.01 (5 %)	0.25	± 0.01 (4 %)
Stress σ_{22lim}; MPa	– 0.05	± 0.01 (20 %)	– 0.09	± 0.01 (11 %)	– 0.14	± 0.01 (7 %)
Strain ε_{22lim}	– 0.010	± 0.002 (20 %)	– 0.007	± 0.001 (14 %)	– 0.007	± 0.001 (14 %)

Table S4. Mechanical properties of PU foams in compression parallel to axis OX_3 (the experimental standard deviation ± s and coefficient of variations v, in %).

Property	Number of a block					
	1		2		3	
Density ρ; kg/m³	32	± 0.2 (1 %)	55	± 0.1 (0 %)	75	± 0.9 (1 %)
Modulus E_3; MPa	10.4	± 0.5 (5 %)	19.4	± 0.3 (2 %)	28.7	± 1.1 (4 %)
Poisson´s ratio ν_{31}	0.52	± 0.02 (4 %)	0.52	± 0.01 (2 %)	0.44	± 0.01 (2 %)
Poisson´s ratio ν_{32}	0.44	± 0.02 (5 %)	0.49	± 0.03 (6 %)	0.38	± 0.01 (3 %)
Stress σ_{33lim}; MPa	– 0.17	± 0.02 (12 %)	– 0.32	± 0.05 (16 %)	– 0.37	± 0.03 (8 %)
Strain ε_{33lim}	– 0.016	± 0.002 (13 %)	– 0.017	± 0.002 (12 %)	– 0.013	± 0.002 (15 %)

Table S5. Mechanical properties of PU foams in shear (the experimental standard deviation ± s and coefficient of variations v, in %).

Property	Number of a block					
	1		2		3	
Plane X_1OX_2						
Density ρ; kg/m³	36	± 0.6 (2 %)	57	± 0.8 (1 %)	76	± 2.0 (3 %)
Modulus G_{12}; MPa	1.8	± 0.5 (28 %)	3.9	± 0.7 (18 %)	7.1	± 0.6 (8 %)
Stress τ_{12lim}; MPa	0.03	± 0.00 (0 %)	0.07	± 0.01 (14 %)	0.18	± 0.01 (6 %)
Strain ε_{12lim}	0.008	± 0.001 (13 %)	0.009	± 0.002 (22 %)	0.013	± 0.002 (15 %)
Plane X_1OX_3						
Density ρ; kg/m³	37	± 0.6 (2 %)	54	± 0.9 (2 %)	73	± 0.3 (0 %)
Modulus G_{13}; MPa	2.3	± 0.5 (22 %)	5.0	± 0.3 (6 %)	9.3	± 0.5 (5 %)
Stress τ_{13lim}; MPa	0.038	± 0.00 (0 %)	0.072	± 0.01 (14 %)	0.158	± 0.01 (6 %)
Strain ε_{13lim}	0.008	± 0.001 (13 %)	0.010	± 0.001 (10 %)	0.01	± 0.001 (10 %)
Plane X_2OX_3						
Density ρ; kg/m³	36	± 0.3 (1 %)	55	± 0.4 (1 %)	73	± 2.3 (3 %)
Modulus G_{23}; MPa	2.9	± 0.6 (21 %)	4.9	± 1.4 (29 %)	8.6	± 1.7 (20 %)
Stress τ_{23lim}; MPa	0.05	± 0.01 (20 %)	0.09	± 0.02 (27 %)	0.15	± 0.03 (20 %)
Strain ε_{23lim}	0.01	± 0.00 (0 %)	0.01	± 0.00 (0 %)	0.01	± 0.00 (0 %)

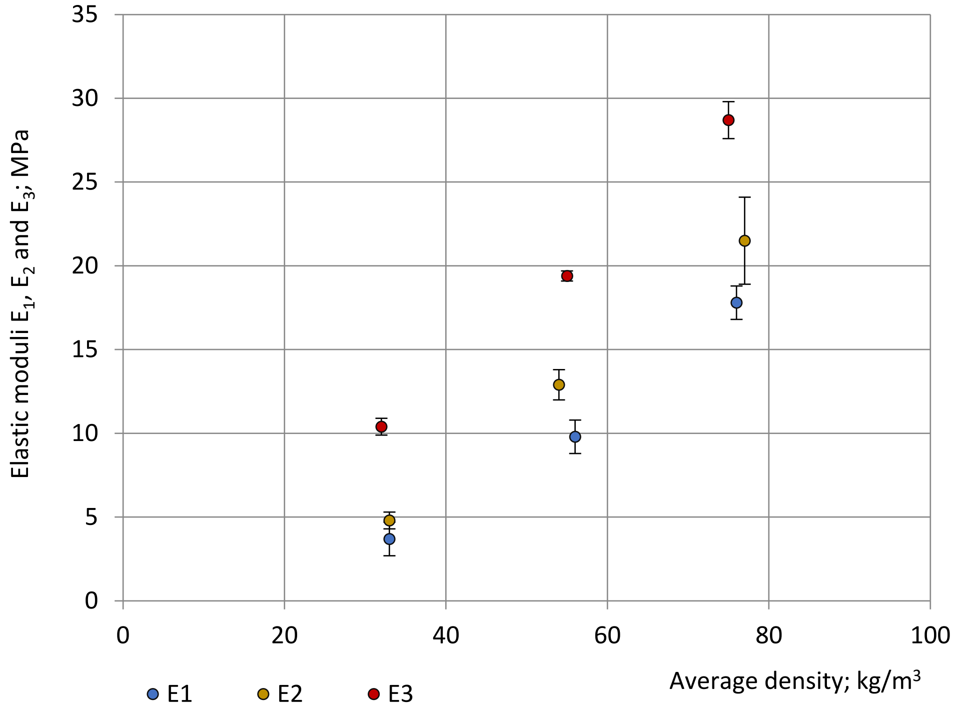

Figure S6. Moduli E_1, E_2 and E_3 of PU foams in the bocks of average density 34 kg/m^3, 55 kg/m^3 and 75 kg/m^3.

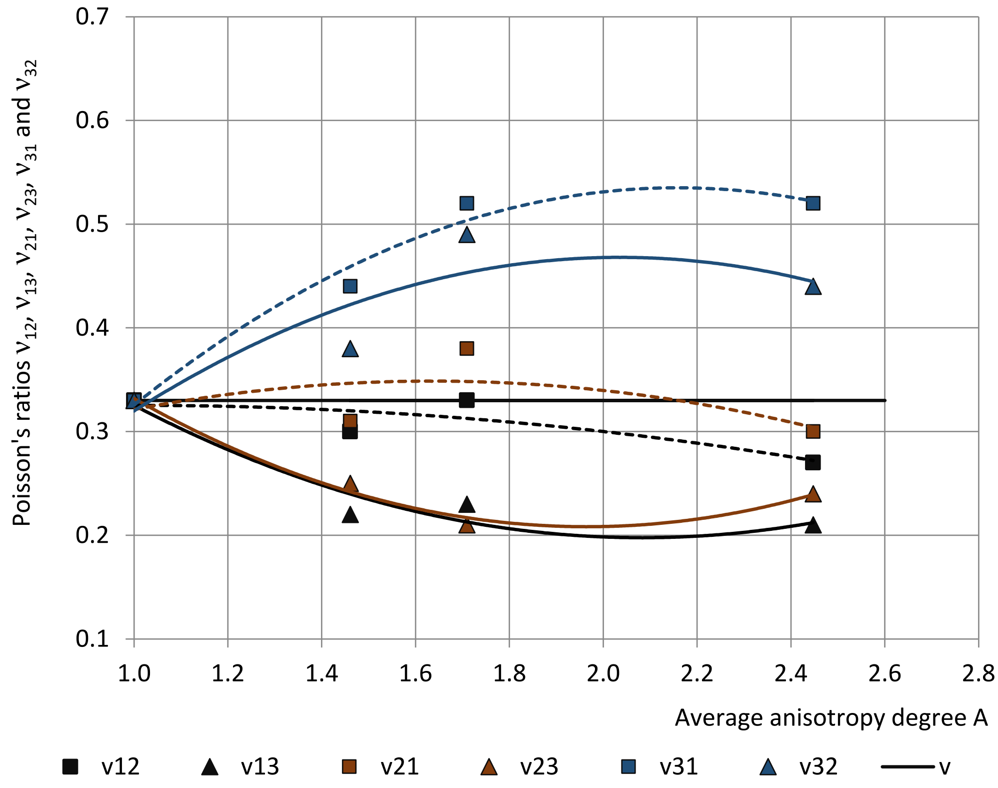

Figure S7. Poisson's ratios ν_{12}, ν_{13}, ν_{21}, ν_{23}, ν_{31} and ν_{32} of PU foams in dependence of average anisotropy degree A (ν = 0.33 is Poisson's ratio of isotropic PU foams).

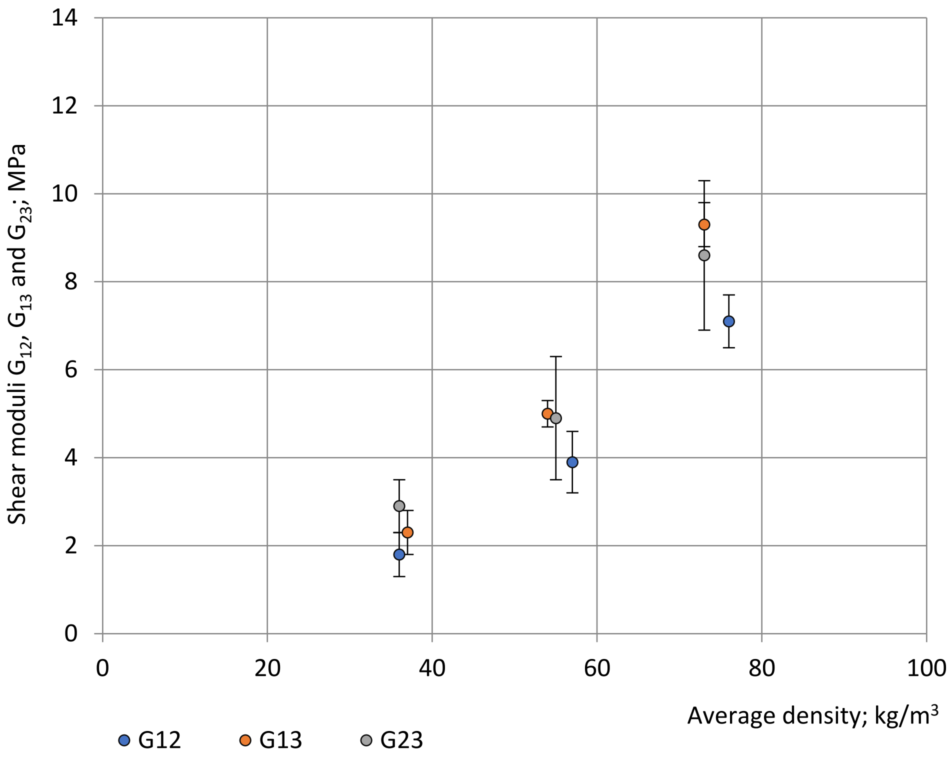

Figure S8. Shear moduli G_{12}, G_{13} and G_{23} of PU foams in the bocks of average density 34 kg/m^3, 55 kg/m^3 and 75 kg/m^3.

Table S6. The calculated virtual stresses at different densities of PU foams.

**Average density; kg/m³**	**Hydrostatic pressure σ_{HP}; MPa**	**Shear stress**	
		**τ′; MPa**	**τ; MPa**
34	– 0.045	0.042	0.030
54	– 0.085	0.080	0.072
75	– 0.135	0.152	0.180

Analysis of uncertainties

1.Ratio f_1

The ratio f_1 is a function of four uncorrelated random variables ν_{13}, ν_{23}, E_1 and E_2:

$$f_1 = \frac{\nu''}{E} = \frac{\nu_{13} + \nu_{23}}{E_1 + E_2}. \quad (S10)$$

For each variable n = 4 statistically independent observations were made (Tables S2 and S3 of Supplementary Materials). For the Type A evaluation of the standard uncertainty, the estimates of ν_{13}, ν_{23}, E_1 and E_2 were calculated as arithmetic means of the individual observed values [28-31]:

$$\bar{\nu}_{13} = \frac{1}{4}\sum_{j=1}^{4} \nu_{13j}\,,\ \bar{\nu}_{23} = \frac{1}{4}\sum_{j=1}^{4} \nu_{23j},$$

$$\bar{E}_1 = \frac{1}{4}\sum_{j=1}^{4} E_{1j} \ \text{and}\ \bar{E}_2 = \frac{1}{4}\sum_{j=1}^{4} E_{2j}. \quad (S11)$$

(For rigid PU foams tested in a certified testing laboratory, the Type B uncertainties are significantly smaller than the Type A uncertainties and are therefore not included in the uncertainty budget.) Based on the previous experimental experience, it is assumed that

the underlying probability distribution for random variables ν_{13}, ν_{23}, E_1 and E_2 is normal. When a number of distributions of whatever form are combined, it can be shown that, apart from in exceptional cases, the resulting probability distribution tends to the normal form in accordance with the Central Limit Theorem. In practice, if three or more distributions of similar magnitude are present, they will usually combine to form a reasonable approximation to the normal distribution. It is assumed further that the values of function f_1 follow a normal distribution.

The estimate of f_1 was determined from estimates of the input quantities as:

$$f_1 = \frac{\bar{\nu}_{13} + \bar{\nu}_{23}}{\bar{E}_1 + \bar{E}_2}. \quad (S12)$$

The experimental variances $Var(q) = s^2(q)$ of the $n = 4$ individual observed values of input quantities were calculated as:

$$Var(\nu_{13}) = \frac{1}{4-1}\sum_{j=1}^{4}\left(\nu_{13j} - \bar{\nu}_{13}\right)^2,\ Var(\nu_{23}) = \frac{1}{4-1}\sum_{j=1}^{4}\left(\nu_{23j} - \bar{\nu}_{23}\right)^2,$$

$$Var(E_1) = \frac{1}{4-1}\sum_{j=1}^{4}\left(E_{1j} - \bar{E}_1\right)^2 \text{ and } Var(E_2) = \frac{1}{4-1}\sum_{j=1}^{4}\left(E_{2j} - \bar{E}_2\right)^2, \quad (S13)$$

where $s(q)$ is the experimental standard deviation of input quantity q. Since the experimental variance of the mean $\bar{q}$ of an input quantity q equals

$Var(\bar{q}) = Var(q)/n$, then:

$$Var(\bar{\nu}_{13}) = \frac{Var(\nu_{13})}{4} = \frac{[s(\nu_{13})]^2}{4},\quad Var(\bar{\nu}_{23}) = \frac{Var(\nu_{23})}{4} = \frac{[s(\nu_{23})]^2}{4},$$

$$Var(\bar{E}_1) = \frac{Var(E_1)}{4} = \frac{[s(E_1)]^2}{4} \text{ and } Var(\bar{E}_2) = \frac{Var(E_2)}{4} = \frac{[s(E_2)]^2}{4}. \quad (S14)$$

Then the experimental standard uncertainty of the mean was calculated for the input quantities as:

$$u(\bar{\nu}_{13}) = \sqrt{Var(\bar{\nu}_{13})} = \sqrt{\frac{Var(\nu_{13})}{4}},\ u(\bar{\nu}_{23}) = \sqrt{Var(\bar{\nu}_{23})} = \sqrt{\frac{Var(\nu_{23})}{4}},$$

$$u(\bar{E}_1) = \sqrt{Var(\bar{E}_1)} = \sqrt{\frac{Var(E_1)}{4}} \text{ and } u(\bar{E}_2) = \sqrt{Var(\bar{E}_2)} = \sqrt{\frac{Var(E_2)}{4}}. \quad (S15)$$

The function f_1 is linear with respect to the variables ν_{13} and ν_{23}, where $0 < \nu_{13}, \nu_{23} < 1.0$ as Poisson's ratios of a foams material. The function f_1 is a power function with respect to the quantities E_1 and E_2, where $E_1, E_2 > 0$ as elastic moduli of a foams material. The function f_1 is considered to be sufficiently smooth and at small uncertainties its variance can be calculated by the delta method (relying on expansion of the function into the Taylor series) [28-31]:

$$Var(f_1) \approx \left(\frac{\partial f_1}{\partial \bar{\nu}_{13}}\right)^2 Var(\bar{\nu}_{13}) + \left(\frac{\partial f_1}{\partial \bar{\nu}_{23}}\right)^2 Var\bar{\nu}_{23} + \left(\frac{\partial f_1}{\partial \bar{E}_1}\right)^2 Var(\bar{E}_1) + \left(\frac{\partial f_1}{\partial \bar{E}_2}\right)^2 Var(\bar{E}_2). \quad (S16)$$

The sensitivity coefficients c (partial derivatives) associated with the input estimates $\bar{\nu}_{13}, \bar{\nu}_{23}, \bar{E}_1$ and $\bar{E}_2$ were calculated:

$$c_1 = \frac{\partial f_1}{\partial \bar{\nu}_{13}} = \frac{1}{\bar{E}_1 + \bar{E}_2},\ c_2 = \frac{\partial f_1}{\partial \bar{\nu}_{23}} = \frac{1}{\bar{E}_1 + \bar{E}_2},$$

$$c_3 = \frac{\partial f_1}{\partial \bar{E}_1} = -\frac{\bar{\nu}_{13} + \bar{\nu}_{23}}{(\bar{E}_1 + \bar{E}_2)^2} \text{ and } c_4 = \frac{\partial f_1}{\partial \bar{E}_2} = -\frac{\bar{\nu}_{13} + \bar{\nu}_{23}}{(\bar{E}_1 + \bar{E}_2)^2}. \quad (S17)$$

It can be seen that $c_1 = c_2$ and $c_3 = c_4$, then:

$$Var(f_1) \approx c_1{}^2[Var(\bar{\nu}_{13}) + Var\bar{\nu}_{23}] + c_3{}^2[Var(\bar{E}_1) + Var\bar{E}_2] =$$

$$= \left(\frac{1}{\bar{E}_1 + \bar{E}_2}\right)^2 [Var(\bar{\nu}_{13}) + Var\bar{\nu}_{23}] + \left[-\frac{\bar{\nu}_{13} + \bar{\nu}_{23}}{(\bar{E}_1 + \bar{E}_2)^2}\right]^2 [Var(\bar{E}_1) + Var\bar{E}_2]. \quad (S18)$$

The combined standard uncertainty of the output estimate f_1 of $N = 4$ input quantities equals:

$$u_c^2(f_1) = Var(f_1) = \sum_{i=1}^{N} u_i^2(f_1) = \sum_{i=1}^{N} c_i^2 u^2(x_i), \quad (S19)$$

$$u_c(f_1) = \sqrt{Var(f_1)}. \quad (S20)$$

Then the range of f_1 values was calculated as $f_{1min} \le f_1 \le f_{1max}$, where $f_{1min} = f_1 - u_c(f_1)$ and $f_{1max} = f_1 + u_c(f_1)$.

The expanded uncertainty is $U(f_1) = ku_c(f_1)$, where k is a coverage factor. The effective degrees of freedom ν_{eff} of the combined standard uncertainty $u_c(f_1)$ associated with the output estimate of f_1 were estimated from the Welch – Satterthwaite formula:

$$\nu_{eff} = \frac{u_c^4(f_1)}{\sum_{i=1}^{N=4} \frac{[u_i(f_1)]^4}{\nu_i}}, \text{ where } \nu_i = n_i - 1. \quad (S21)$$

With ν_{eff} known, the coverage factor was determined from numerical tables, based on a t-distribution evaluated for a coverage probability 95.45 % [28-31], Table S7. Then the range of f_1 values was calculated as $f_{1min} \le f_1 \le f_{1max}$, where $f_{1min} = f_1 - U(f_1)$ and $f_{1max} = f_1 + U(f_1)$.

Table S7. The effective degrees of freedom and coverage factor.

Average density; kg/m³	Effective degrees of freedom ν_{eff}	Coverage factor k
34	7.67	2.40
55	7.17	2.43
75	8.64	2.35

2.Ratio f_2

The ratio f_2 is a function of three uncorrelated random variables ν_{31}, ν_{32} and E_3:

$$f_2 = \frac{\nu'}{E'} = \frac{\nu_{31} + \nu_{32}}{2E_3}. \quad (S22)$$

For each variable four statistically independent observations were made (Table S4 of Supplementary Materials). The arithmetic mean of the individual measured values equals:

$$\bar{\nu}_{31} = \frac{1}{4}\sum_{j=1}^{4} \nu_{31j}, \ \bar{\nu}_{32} = \frac{1}{4}\sum_{j=1}^{4} \nu_{32j} \text{ and } \bar{E}_3 = \frac{1}{4}\sum_{j=1}^{4} E_{3j}. \quad (S23)$$

Based on the previous experimental experience, it was assumed that the underlying probability distribution for ν_{31}, ν_{32} and E_3 is normal. It is assumed further that the values of function f_2 follow normal distribution.

The estimate of f_1 was determined from estimates of the input quantities as:

$$f_2 = \frac{\bar{\nu}_{31} + \bar{\nu}_{32}}{\bar{E}_3}. \quad (S24)$$

The experimental variances of the individual observed values were calculated as:

$$Var(\nu_{31}) = \frac{1}{4-1}\sum_{j=1}^{4}\left(\nu_{31j} - \bar{\nu}_{31}\right)^2, \ Var(\nu_{32}) = \frac{1}{4-1}\sum_{j=1}^{4}\left(\nu_{32j} - \bar{\nu}_{32}\right)^2 \text{and}$$

$$Var(E_3) = \frac{1}{4-1}\sum_{j=1}^{4}\left(E_{3j} - \bar{E}_3\right)^2. \quad (S25)$$

Since the experimental variance of the mean $Var(\bar{q}) = Var(q)/n$, then:

$$Var(\bar{\nu}_{31}) = \frac{Var(\nu_{31})}{4} = \frac{[s(\nu_{31})]^2}{4}, \quad Var(\bar{\nu}_{32}) = \frac{Var(\nu_{32})}{4} = \frac{[s(\nu_{32})]^2}{4},$$

$$Var(\bar{E}_3) = \frac{Var(E_3)}{4} = \frac{[s(E_3)]^2}{4}. \quad (S26)$$

Then the experimental standard uncertainty of the mean was calculated for the input quantities as:

$$u(\bar{\nu}_{31}) = \sqrt{Var(\bar{\nu}_{31})} = \sqrt{\frac{Var(\nu_{31})}{4}},\ u(\bar{\nu}_{32}) = \sqrt{Var(\bar{\nu}_{32})} = \sqrt{\frac{Var(\nu_{32})}{4}} \text{ and}$$

$$u(\bar{E}_3) = \sqrt{Var(\bar{E}_3)} = \sqrt{\frac{Var(E_3)}{4}}. \quad (S27)$$

The function f_2 is linear with respect to the variables ν_{31} and ν_{32} ($0 < \nu_{31}$ and $\nu_{32} < 1.0$ as Poisson's ratios of a foams material) and f_2 is a power function with respect to the quantities E_3 (E_3 as elastic modulus of a foams material). The function f_2 is considered to be sufficiently smooth and at small uncertainties its variance was calculated as:

$$Var(f_2) \approx \left(\frac{\partial f_2}{\partial \bar{\nu}_{31}}\right)^2 Var(\bar{\nu}_{31}) + \left(\frac{\partial f_2}{\partial \bar{\nu}_{32}}\right)^2 Var\bar{\nu}_{32} + \left(\frac{\partial f_2}{\partial \bar{E}_3}\right)^2 Var(\bar{E}_3). \quad (S28)$$

The sensitivity coefficients associated with the input estimates $\bar{\nu}_{31}, \bar{\nu}_{32}$ and $\bar{E}_3$ were calculated:

$$c_1 = \frac{\partial f_2}{\partial \bar{\nu}_{31}} = \frac{1}{\bar{E}_3},\ c_2 = \frac{\partial f_2}{\partial \bar{\nu}_{32}} = \frac{1}{\bar{E}_3} \text{ and } c_3 = \frac{\partial f_2}{\partial \bar{E}_3} = -\frac{\bar{\nu}_{31} + \bar{\nu}_{32}}{(\bar{E}_3)^2}. \quad (S29)$$

It can be seen, that $c_1 = c_2$, then:

$$Var(f_2) \approx c_1^2[Var(\bar{\nu}_{31}) + Var\bar{\nu}_{32}] + c_3^2 Var(\bar{E}_3) =$$

$$= \left(\frac{1}{\bar{E}_3}\right)^2 [Var(\bar{\nu}_{31}) + Var\bar{\nu}_{32}] + \left[-\frac{\bar{\nu}_{31} + \bar{\nu}_{32}}{(\bar{E}_3)^2}\right]^2 Var(\bar{E}_3). \quad (S30)$$

The combined standard uncertainty of the function f_1 equals $u_c(f_2) = \sqrt{Var(f_2)}$. Then the range of f_2 values was calculated as $f_{2min} \le f_2 \le f_{2max}$, where $f_{2min} = f_2 - u_c(f_2)$ and $f_{2max} = f_2 + u_c(f_2)$.

The expanded uncertainty equals $U(f_2) = ku(f_2)$, where k is a coverage factor. The effective degrees of freedom ν_{eff} of the combined standard uncertainty $u_c(\varepsilon_t)$ associated with the output estimate of f_2 were estimated from the Welch – Satterthwaite formula, equation (S21). With ν_{eff} known, the coverage factor was determined from numerical tables, based on a t-distribution evaluated for a coverage probability 95.45 %, Table S8. Then the range of f_2 values was calculated as $f_{1min} \le f_1 \le f_{1max}$, where $f_{2min} = f_2 - U(f_2)$ and $f_{2max} = f_2 + U(f_2)$.

Table S8. The effective degrees of freedom and coverage factor.

Average density; kg/m³	Effective degrees of freedom ν_{eff}	Coverage factor k
34	5.30	2.65
55	5.28	2.70
75	4.25	2.83

3.Modulus G

The modulus G is a function of four uncorrelated random variables E_1, E_2, ν_{12}, and ν_{21}:

$$G = \frac{\frac{1}{2}(E_1 + E_2)}{2\left[1 + \frac{1}{2}(\nu_{12} + \nu_{21})\right]} = \frac{E_1 + E_2}{4 + 2(\nu_{12} + \nu_{21})}. \quad (S31)$$

For each variable four statistically independent observations were made (Tables S2 and S3 of Supplementary Materials). For the Type A evaluation of the standard uncertainty, the estimates of E_1, E_2, ν_{12}, and $\nu21$ were calculated as arithmetic mean of the individual observed values:

$$\bar{E}_1 = \frac{1}{4}\sum_{j=1}^{4}(E_1)_j,\ \bar{E}_2 = \frac{1}{4}\sum_{j=1}^{4}(E_2)_j,$$

$$\bar{\nu}_{12} = \frac{1}{4}\sum_{j=1}^{4}(\nu_{12})_j,\ \bar{\nu}_{21} = \frac{1}{4}\sum_{j=1}^{4}(\nu_{21})_j. \quad (S32)$$

Based on the previous experimental experience, it is assumed that the underlying probability distribution for random variables ν_{13}, ν_{23}, E_1 and E_2 is normal. It is assumed further that the values of function G follow normal distribution.

The estimate of G was determined from estimates of the input quantities as:

$$G = \frac{\bar{E}_1 + \bar{E}_2}{2[1 + (\bar{\nu}_{12} + \bar{\nu}_{21})]}. \quad \text{(S33)}$$

The experimental variances of the individual observed values were calculated as:

$$\mathrm{Var}(E_1) = \frac{1}{4-1}\sum_{j=1}^{4}\left(E_{1j} - \bar{E}_1\right)^2,\ \mathrm{Var}(E_2) = \frac{1}{4-1}\sum_{j=1}^{4}\left(E_{2j} - \bar{E}_2\right)^2,$$

$$\mathrm{Var}(\nu_{12}) = \frac{1}{4-1}\sum_{j=1}^{4}\left(\nu_{12j} - \bar{\nu}_{12}\right)^2, \text{ and } \mathrm{Var}(\nu_{21}) = \frac{1}{4-1}\sum_{j=1}^{4}\left(\nu_{21j} - \bar{\nu}_{21}\right)^2. \quad \text{(S34)}$$

Since the experimental variance of the mean $\mathrm{Var}(\bar{q}) = \mathrm{Var}(q)/n$, then:

$$\mathrm{Var}(\bar{E}_1) = \frac{\mathrm{Var}(E_1)}{4} = \frac{[s(E_1)]^2}{4},\ \mathrm{Var}(\bar{E}_2) = \frac{\mathrm{Var}(E_2)}{4} = \frac{[s(E_2)]^2}{4},$$

$$\mathrm{Var}(\bar{\nu}_{12}) = \frac{\mathrm{Var}(\nu_{12})}{4} = \frac{[s(\nu_{12})]^2}{4} \text{ and } \mathrm{Var}(\bar{\nu}_{21}) = \frac{\mathrm{Var}(\nu_{21})}{4} = \frac{[s(\nu_{21})]^2}{4}.$$

(S35)

Then the experimental standard uncertainty of the mean was calculated for the input quantities as:

$$u(\bar{E}_1) = \sqrt{\mathrm{Var}(\bar{E}_1)} = \sqrt{\frac{\mathrm{Var}(E_1)}{4}},\ u(\bar{E}_2) = \sqrt{\mathrm{Var}(\bar{E}_2)} = \sqrt{\frac{\mathrm{Var}(E_2)}{4}},$$

$$u(\bar{\nu}_{12}) = \sqrt{\mathrm{Var}(\bar{\nu}_{12})} = \sqrt{\frac{\mathrm{Var}(\nu_{12})}{4}} \text{ and } u(\bar{\nu}_{21}) = \sqrt{\mathrm{Var}(\bar{\nu}_{21})} = \sqrt{\frac{\mathrm{Var}(\nu_{21})}{4}}. \quad \text{(S36)}$$

The function G is linear with respect to the quantities E_1 and E_2 (E_1, $E_2 > 0$ as elastic moduli of a foams material) and it is a power function with respect to the quantities ν_{12} and ν_{21} ($0 < \nu_{12}$, $\nu_{21} < 1.0$ as Poisson's ratios of a foams material). The function G is considered to be sufficiently smooth and at small uncertainties its variance can be calculated by the delta method (relying on expansion of the function into the Taylor series) [28-31]:

$$\mathrm{Var}(G) \approx \left(\frac{\partial G}{\partial \bar{E}_1}\right)^2 \mathrm{Var}(\bar{E}_1) + \left(\frac{\partial G}{\partial \bar{E}_2}\right)^2 \mathrm{Var}(\bar{E}_2) + \left(\frac{\partial G}{\partial \bar{\nu}_{12}}\right)^2 \mathrm{Var}(\bar{\nu}_{12}) + \left(\frac{\partial G}{\partial \bar{\nu}_{21}}\right)^2 \mathrm{Var}\bar{\nu}_{21}.$$

(S37)

The sensitivity coefficients c associated with the input estimates $\bar{\nu}_{12}, \bar{\nu}_{21}, \bar{E}_1$ and $\bar{E}_2$ were calculated:

$$c_1 = \frac{\partial G}{\partial \bar{E}_1} = \frac{1}{4\left[1 + \frac{1}{2}(\bar{\nu}_{12} + \bar{\nu}_{21})\right]},\ c_2 = \frac{\partial G}{\partial \bar{E}_2} = \frac{1}{4\left[1 + \frac{1}{2}(\bar{\nu}_{12} + \bar{\nu}_{21})\right]},$$

$$c_3 = \frac{\partial G}{\partial \bar{\nu}_{12}} = -\frac{\bar{E}_1 + \bar{E}_2}{8\left[1 + \frac{1}{2}(\bar{\nu}_{12} + \bar{\nu}_{21})\right]^2} \text{ and } c_4 = \frac{\partial G}{\partial \bar{\nu}_{21}} = -\frac{\bar{E}_1 + \bar{E}_2}{8\left[1 + \frac{1}{2}(\bar{\nu}_{12} + \bar{\nu}_{21})\right]^2}. \quad \text{(S38)}$$

It can be seen that $c_1 = c_2$ and $c_3 = c_4$, then:

$$\mathrm{Var}(G) \approx c_1{}^2[\mathrm{Var}(\bar{E}_1) + \mathrm{Var}(\bar{E}_2)] + c_3{}^2[\mathrm{Var}(\bar{\nu}_{12}) + \mathrm{Var}\bar{\nu}_{21}] =$$

$$= \left\{\frac{1}{4\left[1 + \frac{1}{2}(\bar{\nu}_{12} + \bar{\nu}_{21})\right]}\right\}^2 [\mathrm{Var}(\bar{E}_1) + \mathrm{Var}(\bar{E}_2)] + \left\{\frac{\bar{E}_1 + \bar{E}_2}{8\left[1 + \frac{1}{2}(\bar{\nu}_{12} + \bar{\nu}_{21})\right]^2}\right\}^2 [\mathrm{Var}(\bar{\nu}_{12}) + \mathrm{Var}\bar{\nu}_{21}]. \quad \text{(S39)}$$

The combined standard uncertainty of the function G equals $u_c(G) = s(G) = \sqrt{\mathrm{Var}(G)}$. Then the range of G values was calculated as $G_{min} \le G \le G_{max}$, where $G_{min} = G - u_c(G)$ and $G_{max} = G + u_c(G)$.

Table S9. The strains ε_{ij} at nine variants of independent constants of transtropic PU foams (v is the coefficient of variation, in %).

Average density; kg/m³	Strain component	Ordinal number "n" of a variant of independent constants									Average strain components ε_{ijav}	Standard deviation ± s	v; %
		1	2	3	4	5	6	7	8	9			
34	$\varepsilon_{11(n)}$	– 0.0055	– 0.0052	– 0.0052	– 0.0048	– 0.0045	– 0.0047	– 0.0066	– 0.0063	– 0.0063	– 0.0054	± 0.0008	14
	$\varepsilon_{22(n)}$	– 0.0055	– 0.0052	– 0.0052	– 0.0048	– 0.0045	– 0.0047	– 0.0063	– 0.0063	– 0.0063	– 0.0054	± 0.0007	13
	$\varepsilon_{33(n)}$	– 0.0002	0.0004	– 0.0002	– 0.0002	0.0000	– 0.0002	– 0.0002	0.0004	– 0.0002	0.0000	± 0.0003	111
	$\varepsilon_{23(n)}$	0.0082	0.0082	0.0082	0.0082	0.0082	0.0082	0.0082	0.0082	0.0082	0.0082	0.0000	0
	$\varepsilon_{31(n)}$	0.0082	0.0082	0.0082	0.0082	0.0082	0.0082	0.0082	0.0082	0.0082	0.0082	0.0000	0
	$\varepsilon_{12(n)}$	0.0091	0.0091	0.0091	0.0083	0.0083	0.0083	0.0083	0.0083	0.0083	0.0086	± 0.0004	4
54	$\varepsilon_{11(n)}$	– 0.0014	– 0.0017	– 0.0017	– 0.0016	– 0.0019	– 0.0018	– 0.0010	– 0.0013	– 0.0013	– 0.0015	± 0.0003	21
	$\varepsilon_{22(n)}$	– 0.0014	– 0.0017	– 0.0017	– 0.0016	– 0.0019	– 0.0018	– 0.0013	– 0.0013	– 0.0013	– 0.0015	± 0.0003	17
	$\varepsilon_{33(n)}$	0.0000	– 0.0006	0.0000	0.0000	– 0.0004	0.0000	0.0000	– 0.0006	0.0000	– 0.0002	± 0.0003	171
	$\varepsilon_{23(n)}$	0.0043	0.0043	0.0043	0.0043	0.0043	0.0043	0.0043	0.0043	0.0043	0.0043	0.0000	0
	$\varepsilon_{31(n)}$	0.0043	0.0043	0.0043	0.0043	0.0043	0.0043	0.0043	0.0043	0.0043	0.0043	0.0000	0
	$\varepsilon_{12(n)}$	0.0036	0.0036	0.0036	0.0039	0.0039	0.0039	0.0039	0.0039	0.0039	0.0038	± 0.0001	4
75	$\varepsilon_{11(n)}$	– 0.0009	– 0.0011	– 0.0011	– 0.0010	– 0.0011	– 0.0011	– 0.0008	– 0.0009	– 0.0009	– 0.0010	± 0.0001	13
	$\varepsilon_{22(n)}$	– 0.0009	– 0.0011	– 0.0011	– 0.0010	– 0.0011	– 0.0011	– 0.0009	– 0.0009	– 0.0009	– 0.0010	± 0.0001	11
	$\varepsilon_{33(n)}$	– 0.0003	– 0.0005	– 0.0002	– 0.0003	– 0.0004	– 0.0003	– 0.0003	– 0.0005	– 0.0002	– 0.0003	±0.0001	33
	$\varepsilon_{23(n)}$	0.0024	0.0024	0.0024	0.0024	0.0024	0.0024	0.0024	0.0024	0.0024	0.0024	0.0000	0
	$\varepsilon_{31(n)}$	0.0024	0.0024	0.0024	0.0024	0.0024	0.0024	0.0024	0.0024	0.0024	0.0024	0.0000	0
	$\varepsilon_{12(n)}$	0.0020	0.0020	0.0020	0.0021	0.0021	0.0021	0.0021	0.0021	0.0021	0.0021	± 0.0001	3

Table S10. The relative difference $R_{k(n)}$ between the strains $\varepsilon_{k(n)}$ and the averaged strains ε_{kav}, as well as the summary relative difference $R_{(n)}$ for the six strains.

Average density; kg/m³	Relative difference	Ordinal number "n" of a variant of independent constants								
		1	2	3	4	5	6	7	8	9
34	$R_{1(n)}$	– 0.002	0.053	0.053	0.113	0.168	0.132	– 0.208	– 0.154	– 0.154
	$R_{2(n)}$	– 0.008	0.047	0.047	0.107	0.163	0.126	– 0.161	– 0.161	– 0.161
	$R_{3(n)}$	– 6.241	18.803	– 7.284	– 6.241	2.281	– 6.596	– 6.241	18.803	– 7.284
	$R_{4(n)}$	0.000	0.000	0.000	0.000	0.000	0.000	0.000	0.000	0.000
	$R_{5(n)}$	0.000	0.000	0.000	0.000	0.000	0.000	0.000	0.000	0.000
	$R_{6(n)}$	– 0.059	– 0.059	– 0.059	0.029	0.029	0.029	0.029	0.029	0.029
	$R_{(n)}$	6.310	18.962	7.443	6.491	<u>2.640</u>	6.883	6.639	19.147	7.628
54	$R_{1(n)}$	0.0746	– 0.1218	– 0.1218	– 0.0644	– 0.2608	– 0.2134	0.3668	0.1704	0.1704
	$R_{2(n)}$	0.0944	– 0.0978	– 0.0978	– 0.0417	– 0.2339	– 0.1875	0.1881	0.1881	0.1881
	$R_{3(n)}$	1.1449	– 2.5538	1.1083	1.1449	– 1.6607	1.1171	1.1449	– 2.5538	1.1083
	$R_{4(n)}$	0.0000	0.0000	0.0000	0.0000	0.0000	0.0000	0.0000	0.0000	0.0000
	$R_{5(n)}$	0.0000	0.0000	0.0000	0.0000	0.0000	0.0000	0.0000	0.0000	0.0000
	$R_{6(n)}$	0.0516	0.0516	0.0516	– 0.0258	– 0.0258	– 0.0258	– 0.0258	– 0.0258	– 0.0258
	$R_{(n)}$	1.366	2.825	1.379	1.277	2.181	1.544	1.726	2.938	1.493
75	$R_{1(n)}$	– 0.0009	– 0.0011	– 0.0011	– 0.0010	– 0.0011	– 0.0011	– 0.0008	– 0.0009	– 0.0009
	$R_{2(n)}$	– 0.0009	– 0.0011	– 0.0011	– 0.0010	– 0.0011	– 0.0011	– 0.0009	– 0.0009	– 0.0009
	$R_{3(n)}$	– 0.0003	– 0.0005	– 0.0002	– 0.0003	– 0.0004	– 0.0003	– 0.0003	– 0.0005	– 0.0002
	$R_{4(n)}$	0.0024	0.0024	0.0024	0.0024	0.0024	0.0024	0.0024	0.0024	0.0024
	$R_{5(n)}$	0.0024	0.0024	0.0024	0.0024	0.0024	0.0024	0.0024	0.0024	0.0024
	$R_{6(n)}$	0.0020	0.0020	0.0020	0.0021	0.0021	0.0021	0.0021	0.0021	0.0021
	$R_{(n)}$	0.277	0.651	0.451	<u>0.273</u>	0.630	0.518	0.518	0.750	0.550
